# Gene expression noise in a complex artificial toxin expression system

**DOI:** 10.1371/journal.pone.0227249

**Published:** 2020-01-21

**Authors:** Alexandra Goetz, Andreas Mader, Benedikt von Bronk, Anna S. Weiss, Madeleine Opitz

**Affiliations:** Faculty of Physics and Center for NanoScience, Ludwig-Maximilians-Universität München, Geschwister-Scholl-Platz 1, Munich, Germany; San Jose State University, UNITED STATES

## Abstract

Gene expression is an intrinsically stochastic process. Fluctuations in transcription and translation lead to cell-to-cell variations in mRNA and protein levels affecting cellular function and cell fate. Here, using fluorescence time-lapse microscopy, we quantify noise dynamics in an artificial operon in *Escherichia coli*, which is based on the native operon of ColicinE2, a toxin. In the natural system, toxin expression is controlled by a complex regulatory network; upon induction of the bacterial SOS response, ColicinE2 is produced (*cea* gene) and released (*cel* gene) by cell lysis. Using this ColicinE2-based operon, we demonstrate that upon induction of the SOS response noise of cells expressing the operon is significantly lower for the (mainly) transcriptionally regulated gene *cea* compared to the additionally post-transcriptionally regulated gene *cel*. Likewise, we find that mutations affecting the transcriptional regulation by the repressor LexA do not significantly alter the population noise, whereas specific mutations to post-transcriptionally regulating units, strongly influence noise levels of both genes. Furthermore, our data indicate that global factors, such as the plasmid copy number of the operon encoding plasmid, affect gene expression noise of the entire operon. Taken together, our results provide insights on how noise in a native toxin-producing operon is controlled and underline the importance of post-transcriptional regulation for noise control in this system.

## Introduction

Gene expression noise, the variation of gene expression from cell to cell observed in isogenic populations, arises from stochasticity in biochemical processes [1–4]. Studying simple and often artificial gene expression networks, regulation [5–7] and control [8,9] of gene expression noise as well as noise propagation [10,11] has been investigated both in eukaryotes [8,12–14] and bacteria [15–19]. Noise in gene expression is mostly thought to be deleterious [20], but can be beneficial, if the resulting phenotypic heterogeneity within the isogenic population provides the bacterial population with the flexibility to adapt to fluctuating environments or to respond to external stresses [18,21]. Although, the basic principles of noise generation and propagation in gene expression networks are well understood [22–25], little is known on how noise is controlled in complex regulatory networks, that comprise both chromosomally and plasmid-encoded network components. Here, the expression of genes encoding phenotypic functions such as the production of toxic colicins [26] or antibiotic resistances [27,28] from multi-copy plasmids, is controlled by chromosomally encoded transcriptional and post-transcriptional regulatory units, that are intrinsically exposed to fluctuations in biochemical reaction rates.

**Fig 1:**
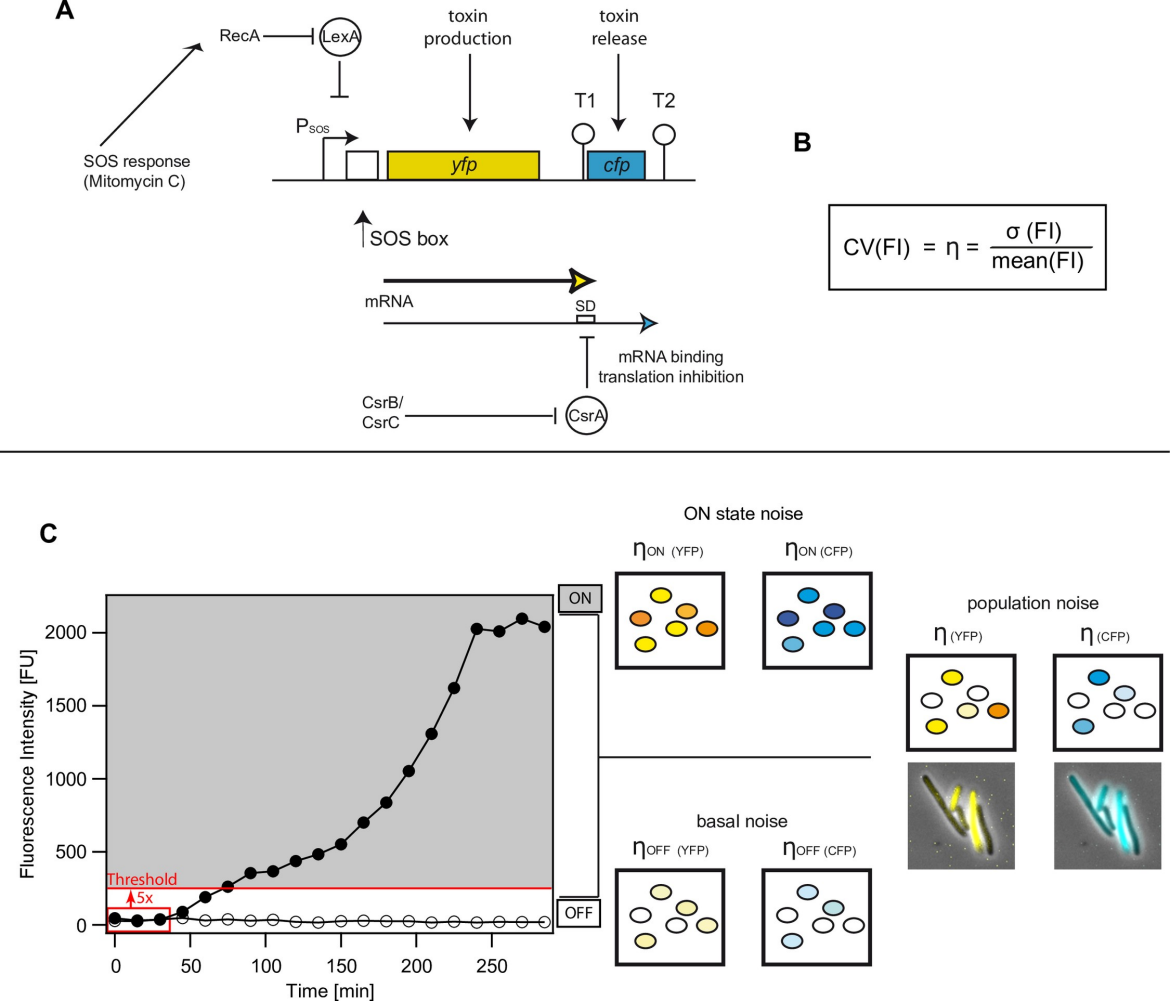
Noise types in the model system. A) Schematic of the gene regulation network of the model operon (adapted from Götz *et al*. [48], ColicinE2 operon). On the reporter plasmid pMO3, present in the S strain (Table 1, S1 Table), the genes *cea* and *cel* of the ColicinE2 operon coding for a toxin (*cea*) and release protein (*cel*) are replaced by genes that code for the fluorescent proteins YFP and CFP respectively. Expression of the operon is repressed by the transcriptional regulator LexA, which is cleaved from the SOS promoter with the help of RecA upon SOS response activation. With release of LexA from the SOS box, transcription of a short mRNA including the *yfp* gene and a long mRNA, additionally including the *cfp* gene takes place. The mRNA binding protein CsrA regulates CFP expression post-transcriptionally by binding to the Shine-Dalgarno (SD) sequence of the long mRNA. The amount of freely available CsrA in the cell is regulated by the sRNAs CsrB and CsrC. B) As a measure for noise we determined the coefficient of variation (CV(FI)). C) Development of fluorescence intensity was obtained for single cells over a time-course of 300 min. t = 0 is the time-point of MitC addition. Two exemplary single cell traces are shown for a cell strongly expressing YFP (ON-state, filled circles) and a non-expressing cell (OFF-state, open circles). Cells are in the ON-state if they overcome a threshold intensity of 5x their initial fluorescence (red line). We distinguish three different types of noise: First, basal noise of cells in the OFF state. Second, the noise of cells in the ON-state and finally, the population noise of all cells. Exemplary microscopy images showing the noise of the entire population are given as color overlay.

In this work, we investigate noise in an artificial toxin expression system that is based on the well-studied ColicinE2 system [29–35] of *Escherichia coli*. The native ColicinE2 toxin expression system constitutes a paradigmatic model to study gene expression noise, as here transcriptional and post-transcriptional regulation mechanisms are combined to control ColicinE2 expression and release. ColicinE2, as many other bacteriocins, is encoded on a multi-copy plasmid and heterogeneously expressed [36–39] from an operon under the control of an SOS promoter [29,40] in response to external stresses. The artificial system investigated here was designed to reflect the regulation mechanisms of the native ColicinE2 operon. In order to enable observation with fluorescence time-lapse microscopy, the sequences for the toxin (*cea*) and lysis gene (*cel*, the gene triggering toxin release) of the native ColicinE2 operon are replaced with a yellow and cyan fluorescent protein (YFP and CFP), respectively. This ColicinE2-based operon is encoded on the pBAD multi-copy reporter plasmid and introduced into the native cells (Materials and methods). Identical to the native ColicinE2 system, upon induction of the SOS response, RecA induces autocleavage of LexA dimers in the *E*. *coli* cell. This permits the production of two mRNAs due to the presence of two transcriptional terminators T1 and T2 (Fig 1A): the short mRNA including *yfp*, and the long mRNA encoding both *yfp* and *cfp* [26]. Translation of the *cfp* gene is inhibited post-transcriptionally by the mRNA-binding protein CsrA that binds to the Shine-Dalgarno Sequence (SD) of the *cfp* gene on the long mRNA [41]. The abundance of CsrA is further regulated by two CsrA-sequestering sRNAs: CsrB and CsrC [42–47].

In this study, we examine gene expression of *yfp* and *cfp* (corresponding to the genes *cea* and *cel* of the native ColicinE2 operon) to investigate, if noise in gene expression differs for the individual genes and how it is affected by transcriptional and post-transcriptional mechanisms. We find population noise in *cel* expression to be significantly higher compared to *cea* expression investigating cells expressing the ColicinE2-based operon from the pBAD multi-copy plasmid (Materials and methods). Furthermore, we find that mutations affecting the transcriptional regulation via the chromosomally encoded repressor LexA do not significantly alter the population noise. In contrast, specific mutations to post-transcriptionally regulating units, such as the global carbon storage regulator CsrA, or sRNAs sequestering CsrA, affect both *cea* and *cel* expression noise in our artificial system.

## Results

### Expression noise in the ColicinE2-based toxin expression system

In order to study gene expression noise in the ColicinE2-based toxin expression system, we analyzed the expression of yellow and cerulean fluorescence proteins (YFP and CFP), using fluorescence time-lapse microscopy, from the reporter plasmid pMO3 in a bacterial strain lacking the original ColicinE2 plasmid, the S strain (Table 1 and S1 Table). We chose to investigate gene expression noise in this reporter strain, as in the native strain carrying the toxin-producing pColE2-P9 plasmid, an ssDNA intermediate of autonomous plasmid replication, constituting an additional CsrA-sequestering element, interferes with ColicinE2 expression dynamics [48] and thus noise generation and control in the pure ColicinE2 operon cannot be investigated. Consequently, to study the influence of certain regulatory elements (transcriptional and post-transcriptional) on gene expression noise in the ColicinE2 operon only, we investigated noise in the S reporter strain in the absence of these ssDNA dynamics.

**Table 1:** Genetic modifications and expected effects on transcription and translation in mutant strains in comparison to the wild-type reporter strain, the S strain. The table summarizes the genetic details and modifications of each strain, states the resulting effects, reports on expected changes in transcription, translation or mRNA decay in each strain. Further strain details can be found in S2 Table and sequence specifications in [48].

Bacterial strain	Genetic modification/information	Effect	Expected change in transcription or translation	Expected effect on mRNA decay
S	Reporter strain	-	-	-
LexA1	Base exchange AT-to-TA in the first SOS box on pMO3	Stronger LexA binding	Reduced transcription events of entire operon	No effects as sequence changes are prior to transcription start
LexA2	Base exchange of CTG-to-CCC in the first SOS box on pMO3	Stronger LexA binding	Reduced transcription events of entire operon	No effects as sequence changes are prior to transcription start
Δ LexA	SOS box sequence deleted on pMO3	No binding of LexA	Constitutive transcription of entire operon (S1 Fig)	No effects as sequence changes are prior to transcription start
CsrA1	Mutation GTC to TGT in the second CsrA binding site in the SD sequence of *cfp* on pMO3	Stronger CsrA binding	Reduction of *cfp* translation events	Sequence changes in RBS of *cfp*. Effects on mRNA decay are expected to be rather small
CsrA2	Mutation AC to TT in the second CsrA binding site in the SD sequence of *cfp* on pMO3	Weaker CsrA binding	Strong increase of *cfp* translation events	Sequence changes in RBS of *cfp*. Effects on mRNA decay are expected to be rather small
CsrB	In-frame replacement of CsrB with kanamycin resistance on the bacterial chromosome	No CsrB	More free CsrA in the bacterial cell. Less *cfp* translation events	-
CsrBC	In-frame replacement of CsrC by kanamycin resistance and of CsrB by chloramphenicol resistance cassette	No CsrB and CsrC	More free CsrA in the bacterial cell. Less *cfp* translation events	-
Δ LexA/CsrA2	SOS box sequence deleted on pMO3, Mutation AC to TT in the second CsrA binding site in the SD sequence of *cfp*	No binding of LexA. Weaker CsrA binding	Constitutive transcription of entire operon (S1 Fig). Strong increase of *cfp* translation events	Sequence changes in RBS of *cfp*. Effects on mRNA decay are expected to be rather small

The pMO3 plasmid, present in the S strain, contains the ColicinE2 operon in which the genes *cea* (toxin production) and *cel* (toxin release) are replaced by gene sequences encoding the YFP and CFP fluorescence proteins (FPs), respectively (Fig 1A) [36]. We chose to use this multi-copy plasmid, as the native plasmid encoding the ColicinE2 operon (pColE2-P9) occurs as a multi-copy plasmid, as well. Hence, the choice of pMO3 mimics the natural behavior (Materials and methods) and allows us to study gene expression noise in the ColicinE2-based operon in a controlled way. In addition, this reporter plasmid carries all genetic sequences relevant for transcriptional and post-transcriptional regulation found in the native ColicinE2 operon, such as the native LexA and CsrA binding sites or the T1 and T2 transcriptional terminators (Fig 1A). Using fluorescence time-lapse microscopy, we monitored fluorescence development over time for YFP and CFP expression and determined the coefficient of variation (CV) for the obtained fluorescence intensities (FI) as a standard measure of noise in gene expression [23] (Fig 1B).

The noisy RecA/LexA regulatory module [49] enables single cells to switch into the toxin-producing state stochastically causing phenotypic heterogeneity within the isogenic bacterial population. In order to study the noise dynamics of YFP and CFP gene expression from the ColicinE2 operon, we performed our experiments in the presence of high external stress levels imposed by the SOS response-inducing agent Mitomycin C (MitC) in the medium (Materials and methods). At these high stress levels, almost all cells switch into the ON state within the first hour and the population reaches a steady state at 270 min (Fig 1C). In the following, we distinguish three different populations and their corresponding noise phenomena: (i) the basal noise of the cells that are not expressing FPs (OFF-state), (ii) the noise of ON cells that are expressing FPs above a 5x threshold (Fig 1C, Materials and methods) to clearly distinguish them from cells in the OFF-state and (iii) the complete population noise (ON and OFF cells) (Fig 1C).

**Fig 2:**
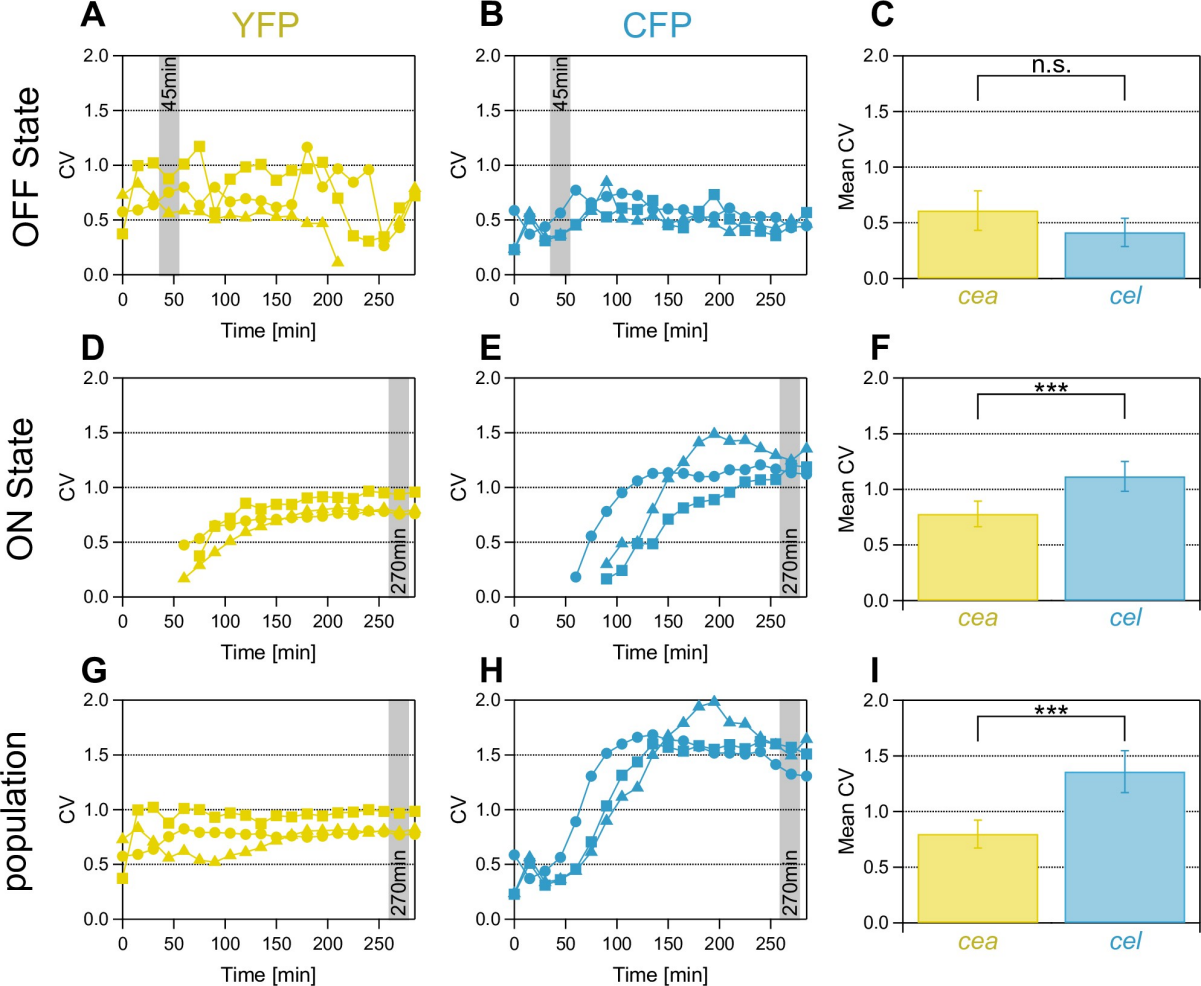
Population noise in CFP is higher than population noise in YFP. Coefficient of variation (CV) over time for all cells, cells in the ON-state and cells in the OFF-state. Squares: 0.1 μg/ml MitC, triangles: 0.25 μg/ml MitC, circles: 0.4 μg/ml MitC. A,D,G) noise in YFP (yellow) expression over time at three different MitC levels (Materials and methods). B, E, H) noise in CFP (blue) expression. The basal noise was quantified as noise of populations for all MitC concentrations at t = 45 min. The production and release noise was determined as the noise at t = 270 min of cells in the ON-state (all MitC levels). The population noise was determined as the noise at t = 270 min for measurements of all stress levels. C) Basal noise for YFP and CFP expression. F) ON state noise (YFP, yellow and CFP, blue). I) Population noise for YFP and CFP expression. Significance levels: n.s. no significant difference, ***: p<0.001. C, F, I present data averaged over all measurements and MitC induction levels. The error bar denotes the 95% confidence interval around the mean of all measurements. Number of replicates N for each bar in C,F,I) is 8, with 310 considered cells in total (I). Detailed information on analyzed cell numbers can be found in the S1 Data file.

In a first experiment, we investigated the noise for these three populations for the S reporter strain. Please find the mean values of the fluorescence intensities (FI) and σ, as well as FI histograms for the three different populations, and the time-development of FI for all MitC concentrations of the population noise in the S1 Fig. We found, that the basal noise of both YFP and CFP is constant over time for all studied MitC concentrations. Further, the CV obtained for the three different MitC induction levels showed no clear difference (Fig 2A and 2B). Thus, we quantified the basal noise over all stress levels and compared them at t = 45 min as at this point, switching into the ON state, due to induction of the SOS response, has not yet started. We found no significant difference between the basal noise of YFP and CFP with 0.61±0.18 and 0.41±0.13, respectively (Fig 2C, the error denotes the 95% confidence interval around the mean of all measurements). These basal noise values are similar to noise levels obtained for cells grown in the absence of the inducer MitC (S2 Fig).

Investigating the noise of cells in the ON-state, we found that it increased over time and finally saturated for both YFP and CFP (Fig 2D and 2E) for all stress levels. This is in accordance with the increasing standard deviation of fluorescence intensity of the ON-population (σ_(FITON)_) (S3 Fig). At later time-points, a large fraction of cells expressed YFP and CFP at high levels compared to early time-points, where gene expression had only started in those cells. Again, the data showed no considerable difference for the three MitC concentrations. Consequently, we quantified the noise of the ON cells over all data obtained for these MitC concentrations at t = 270 min, representing the final noise of toxin production and release (Fig 2F). Interestingly, the noise in toxin production (YFP ON-state, Fig 2F) was not significantly different from the basal noise (YFP OFF-state, Fig 2C). This can be explained as follows: the increase in transcription rate upon SOS induction leads both to an increased σ and mean (FI), hence, the ratio σ/mean (= CV) stays approximately constant (S3 Fig). In contrast, the mean CFP noise for cells in the ON-state increased and now exceeded the YFP noise with a CV of 1.12±0.13 and 0.78±0.11, respectively (Fig 2F).

Finally, we determined the population noise as the noise of the entire population that includes both ON and OFF cells over time (Fig 2G and 2H) and again found, that there are only small differences between the three stress levels and that at t = 270 min a steady state was reached. Calculating the CV of all MitC levels at 270min (Fig 2I) we obtained similar values as for the noise of cells in the ON-state with noise in CFP expression being higher than noise in YFP expression with a mean CV of 1.36±0.19 and 0.80±0.13, respectively.

To rule out that any observed differences in YFP and CFP expression noise are mainly due to differences in the fluorescence reporters, we repeated our experiments with an S strain mutant carrying plasmid pMO11, that is identical to pMO3, but with *cea* now being exchanged by CFP and *cel* by YFP (S1 Table). For this S_FLIP_ mutant strain, we determined the same noise tendencies as for the S ‘wild-type reporter’ strain, confirming our above-described results (S4 Fig).

### Effect of transcriptional and post-transcriptional regulation on gene expression noise in the ColicinE2-based toxin expression system

We had observed that upon induction of the SOS response population noise in CFP was higher than noise in YFP, demonstrating that gene expression noise is different for the individual genes within the artificial ColicinE2-based operon. However, it was not clear which regulatory elements were responsible for this difference. Consequently, we investigated the impact of several regulatory components on YFP and CFP gene expression noise in our system. In Table 1 the exact genetic changes for each mutant are summarized and the expected changes on transcription and translation or mRNA decay in our ColicinE2-based system are explained.

As we had found that the noise of cells in the ON-state was similar to the noise of the entire S strain population, we concentrate on population noise comparisons in the following analysis. The data on basal noise and noise of cells in the ON-state are given in the S5 Fig. Please find the mean values of the fluorescence intensities (FI) and σ for the population noise of all regulatory mutant strains in S6 Fig. In S7 Fig FI histograms are presented for mutant strains with significant changes in population noise.

**Fig 3:**
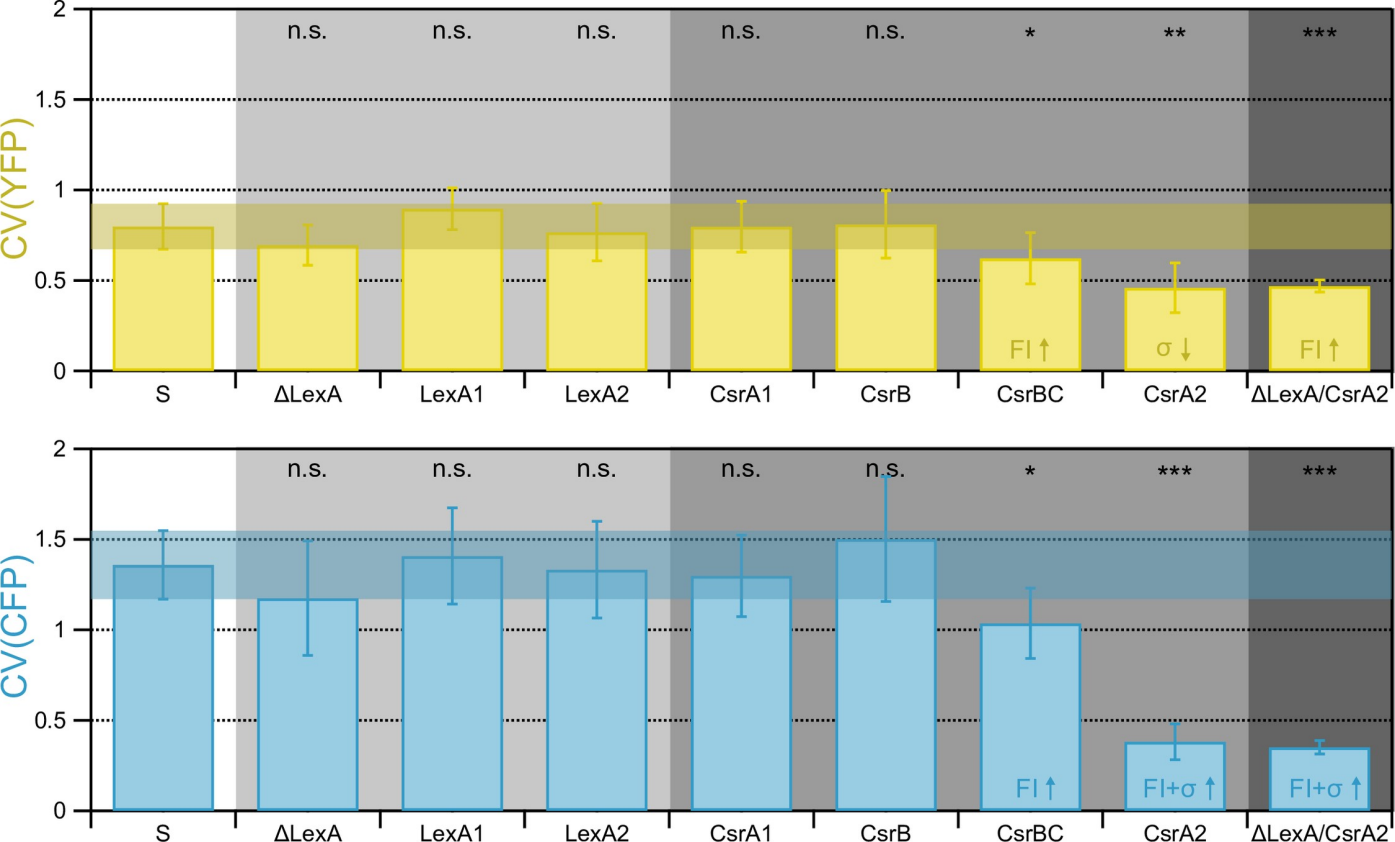
CsrA controls noise in CFP expression and reduces noise of the entire operon. Population noise in YFP and CFP expression are shown in yellow and blue, respectively. Population noise is shown for the S strain as well as several mutant strains (Materials and methods, S1 Table). Background color indicates genetic changes in transcriptional (T, light grey) or post-transcriptional regulation (PT, medium grey), or both (T and PT, dark grey). Transparent colored regions (YFP, yellow and CFP, blue) indicate the CV values (including the corresponding error) of the S strain for better comparability between data sets. Significance levels: n.s. no significant difference; *: p<0.05; **: p<0.01; ***: p<0.001. The FI/σ ↑/↓ up and down arrows indicate the main influence on the observed change in CV. The error bar denotes the 95% confidence interval around the mean of all measurements. Number of replicates N for each strain is S: 8, ΔLexA: 9, LexA1: 11, LexA2: 10, CsrA1: 9, CsrB: 6, CsrBC: 7, CsrA2: 7, ΔLexA/CsrA2: 7. Number of considered cells X: S: 310, ΔLexA: 301, LexA1: 431, LexA2: 382, CsrA1: 434, CsrB: 314, CsrBC: 312, CsrA2: 348, ΔLexA/CsrA2: 247. Detailed information on analyzed cell numbers can be found in the S1 Data file.

As LexA binding alters YFP and CFP expression from the ColicinE2-based operon, we first analysed how variations in the LexA binding site (Materials and methods, Table 1) affect gene expression noise in the ColicinE2-based operon in comparison to the ‘wild-type’ S strain. We found that a mutant strain (ΔLexA, S1 Table) lacking the LexA binding site, which is therefore constitutively expressing the ColicinE2-based operon (S8 Fig), showed a population noise similar to the noise levels observed for the S strain, with 0.70 ± 0.11 for YFP and 1.18 ± 0.32 for CFP expression (Fig 3, S1 Data). As we quantify the population noise at t = 270 min (Fig 2H), the population noise represents the steady state value of the system, when all cells have switched into the toxin-producing state upon SOS response induction with high external stress. Consequently, at this time-point, repression of the operon in the S strain by LexA is absent and the population noise does not differ from the noise values of the unrepressed ColicinE2-based operon in the ΔLexA mutant (Fig 3). We then analysed how variation in LexA binding strength and thus the expression level of the ColicinE2-based operon influences noise in gene expression of the individual genes within the operon. We found that variations in the LexA binding site on the reporter plasmid pMO3 (mutants LexA1 and LexA2, Materials and methods, Table 1, S1 Table) which lead to an increased binding of LexA, did not alter the population noise significantly, neither for YFP or CFP expression (Fig 3, S1 Data).

Now we had learned that differences in LexA binding did not significantly affect population noise in both YFP and CFP expression in our artificial ColicinE2-based toxin expression system. Hence, the in general higher population noise in CFP could not be explained by a regulatory effect imposed by the transcriptional regulator LexA. Therefore, we next assessed the impact of the post-transcriptional regulatory module on gene expression noise in the operon, consisting of the mRNA binding protein CsrA and the two CsrA sequestering sRNAs CsrB and CsrC (Fig 1A). We first quantified the population noise for a mutant strain with increased CsrA binding to the SD sequence present only on the long mRNA transcript (CsrA1, Materials and methods, Table 1, S1 Table). Consequently, the noise in YFP expression should not be affected. Indeed, we obtained a similar population noise value for YFP expression for this mutant as for the S strain (Fig 3, S1 Data). Interestingly, also CFP population noise for the CsrA1 mutant was comparable to the CFP population noise obtained for the S strain (Fig 3, S1 Data**)**. We attribute this to the fact that the native CsrA binding site of the *cel* gene is nearly optimal [50] and our genetic modifications allowed only a slight increase in CsrA binding to the SD-sequence [48] leading to a reduction of the mean FI by a factor of 4, which was accompanied by a reduction of sigma of the same magnitude (S6 Fig). This results in a similar CV value as observed for the S strain. Hence, slightly increasing CsrA binding does not significantly change CFP expression noise.

In the mutant strain CsrB (Table 1), we did not alter any sequence on the reporter plasmid pMO3, but deleted the sRNA gene CsrB in the bacterial genome completely (Materials and methods, S1 Table). As CsrB is sequestering CrsA [42,43], in consequence more CrsA proteins should be available in the bacterial cell, able to repress CFP translation. As such, noise in YFP expression again should be unaffected and we expect noise in CFP expression to be increased due to the higher repressive effect of CsrA. However, we found no significant changes in both YFP and CFP population noise in comparison to the S strain (Fig 3, S1 Data), which can be explained by a compensatory effect of the second CsrA sequestering sRNA CsrC [42].

Consequently, we investigated the mutant strain CsrBC lacking both sRNAs CsrB and CsrC (Materials and methods, Table 1, S1 Table), that should have even more freely available CsrA molecules. We found a small decrease in YFP and CFP population noise to 0.62 ± 0.14 and 1.04 ± 0.19 (Fig 3) that was mainly due to a slight increase in FI. Interestingly, a mutant strain (CsrA2, Table 1) with strongly decreased CsrA binding effectiveness due to changes in the SD sequence of the *cfp* gene on the long mRNA (Materials and methods, S1 Table) also had decreased population noise values both in YFP and CFP expression with 0.46 ± 0.14 and 0.38 ±0.10, respectively (Fig 3). The strong decrease in CFP population noise can be explained by the fact that now CsrA is no longer able to repress *cfp* translation, as due to the genetic modifications introduced in this mutant, CsrA is hardly able to bind to the SD sequence of the *cel* gene [48]. Consequently, *cfp* is highly expressed leading to a strongly increased FI value by a factor of 11 in comparison to the increase in sigma by only a factor of 3, which can explain the observed strong noise reduction in CFP expression (S6 Fig). However, this does not explain the decrease of the population noise in YFP expression that we also observed in the CsrBC mutant.

In order to investigate how combined changes on different levels (transcriptional and post-transcriptional) affect population noise of YFP and CFP, we created the double mutant ΔLexA/CsrA2 (Materials and methods, Table 1, S1 Table). This mutant lacks the LexA binding site required for LexA repression of the operon and CsrA binding is strongly reduced due to changes in the SD—sequence of the *cfp* gene on the long mRNA and thus represents a combination of the mutants ΔLexA and CsrA2. As expected from the results seen for the single ΔLexA or CsrA2 mutants, we found a reduction of the YFP population noise in this mutant with 0.47 ± 0.03 and CFP population noise with 0.35 ± 0.04 (Fig 3).

A further factor that might contribute to the noise dynamics in the ColicinE2 operon is the copy number of the plasmid carrying the ColicinE2 operon [26]. In the native system [51] the operon is expressed from ~ 20 plasmid copies [48,51]. In our artificial system the ColicinE2-based operon is installed on the pMO3 reporter plasmid with ~ 55 copies. Hence, we believe gene expression noise in the native ColicinE2 operon on the pColE2-P9 plasmid of the wild-type strain to be lower than in our artificial system, as high copy numbers as in the artificial system can act to increase noise levels [52]. In accordance with this hypothesis, we found reduced noise values in both YFP and CFP expression (S9 Fig) for a S strain mutant expressing the ColicinE2-based operon from the same reporter plasmid but with a changed origin of replication (S_REP2,_ Materials and methods) reducing the plasmid copy number from ~ 55 for pMO3 (S strain) to ~ 13 (S_REP2_). For S_REP2_ both FI and σ are reduced compared to the S strain values (S9 Fig). However, the reduction of sigma is stronger than the reduction in the FI. For the *cel* gene this is even more pronounced with a reduction in σ by a factor of ~14 and a reduction in FI by a factor of only ~ 7 compared to S strain values. This demonstrates the importance of plasmid copy numbers, as an additional factor in our ColicinE2-based system that contributes to noise generation.

## Discussion

In this study, we investigated noise in gene expression in an artificial toxin-producing operon. We quantified the noise dynamics of this ColicinE2-based operon focusing on differences in the individual genes *yfp* and *cfp* corresponding to the genes *cea* (toxin production) and *cel* (toxin release) in the native operon, respectively. We found that only for cells in the OFF-state the noise in *cea* expression was comparable to noise in *cel*, similar to cells grown in the absence of MitC. In contrast to this basal noise, we found that within the ColicinE2-based operon *cel* gene expression noise of cells in the ON-state, as well as *cel* population noise exceeded the corresponding *cea* noise. Here, at t = 270 min, the ColicinE2 operon is maximally expressed and large amounts of long mRNA (Fig 1) are produced [26]. In addition, with high numbers of long mRNAs present, the same amount of free CsrA proteins within the bacterial cell has to repress a higher number of long mRNAs as compared to the OFF-state. Hence, in the ON-state less free CsrA molecules are available and repression of the *cel* gene (in our case the *cfp* gene) is decreased, which can explain the increase in *cfp* gene expression noise in the ON-state in comparison to the OFF-state.

In addition, we investigated gene expression noise in this ColicinE2-based operon in dependence of two regulatory modules: transcriptional regulation by the repressor LexA [26] and post-transcriptional repression by the Csr system [43,44] comprising the mRNA binding protein CsrA and the two CsrA sequestering sRNAs CsrB and CsrC [42,44]. Starting with mutations in transcription efficiency by changes in LexA repression (ΔLexA, LexA1, LexA2), our data showed that variations in LexA binding did not significantly alter population noise in both YFP and CFP expression (Fig 3). With regard to post-transcriptional regulation, mutants with slightly changed CsrA binding efficiency (CsrA1, CsrB) also showed no significant population noise differences compared to the ‘wild-type reporter’ S strain. However, mutants with strongly altered CsrA binding (CsrA2, ΔLexA/CsrA2) and specifically mutations that increase CsrA abundance in the cell by changing the number of binding partners for CsrA [47] (CsrBC) showed a strong reduction in population noise in CFP, indicating the importance of CsrA availability for *cel* population noise. With regard to the CsrA2 mutant, a further factor might play an important role: The CsrA binding site lies within a stem loop. However, due to the genetic modifications introduced in this mutant strain, this stem loop is no longer able to form and CsrA has a highly reduced binding affinity to the mRNA [48]. Dacheux *et al*., [53] showed that the presence of a stem loop can increase noise in translation in comparison to a control lacking a stem loop structure. Likewise, noise in *cel* gene expression might be strongly reduced in the CsrA2 mutant due to the absence of the stem loop.

**Fig 4:**
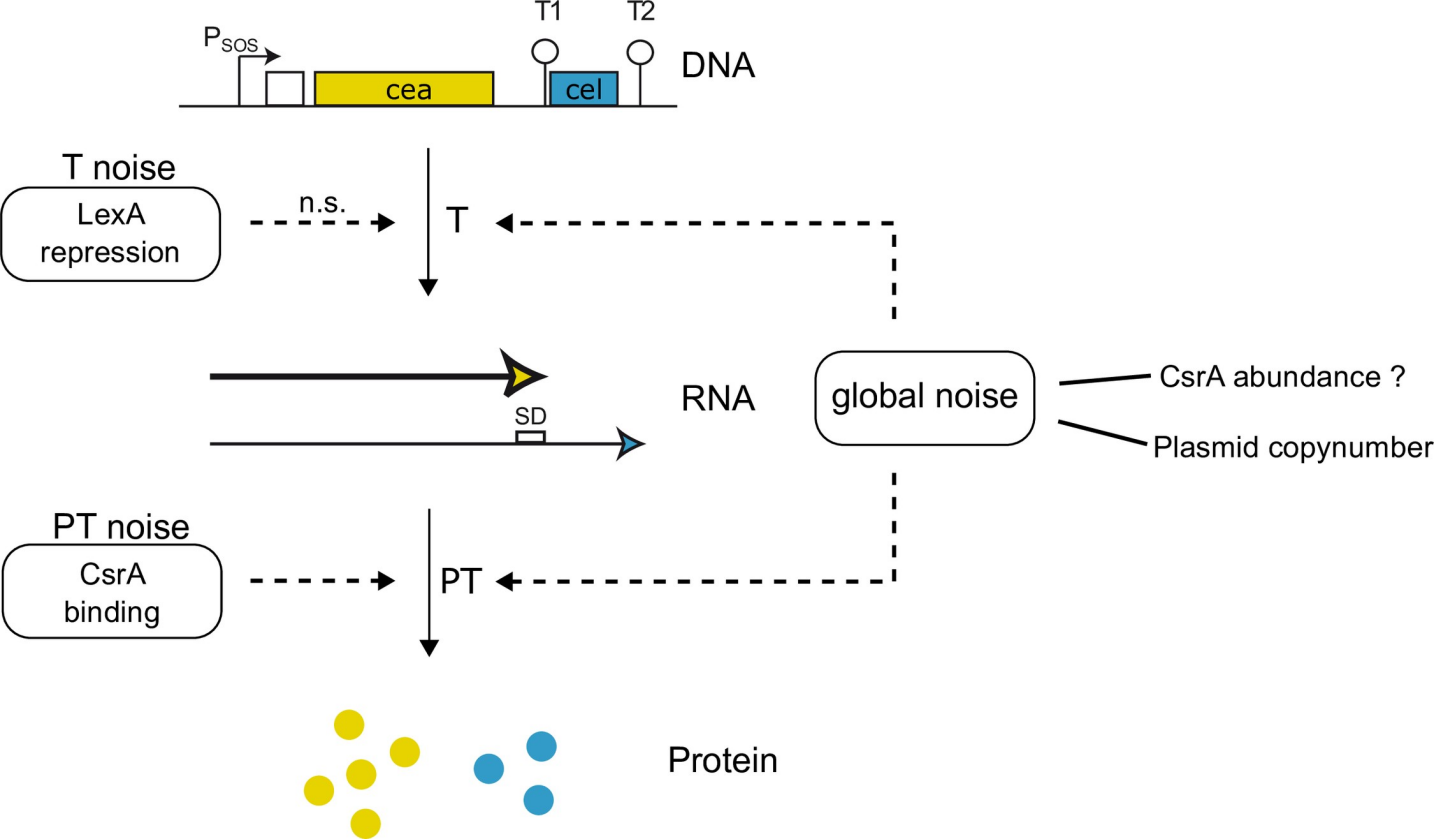
Factors contributing to noise generation in the ColicinE2-based regulatory network. T = transcriptional, PT = post-transcriptional. Solid arrows indicate regulatory mechanisms. Dashed arrows indicate noise influencing elements. n.s.: Variations of LexA binding did not lead to significant changes in population noise. ?: Question mark denotes that our data indicate that CsrA could act either direct or indirect as a global factor affecting population noise.

Unexpectedly, we also found a significant reduction of population noise in YFP expression for the CsrA2, ΔLexA/CsrA2 and CsrBC mutants (Fig 3). This leads us to hypothesize that CsrA also acts as a global regulation factor of gene expression noise in our ColicinE2-based operon. In a study by Bar-Even *et al*., [54] it was nicely shown, that global factors can strongly affect noise generation at all steps of gene expression: transcription as well as translation. The CsrA protein, whose concentration of freely available molecules is either directly or indirectly affected by our genetic changes in the CsrB, CsrB/C or CsrA2 mutant strains, is a highly abundant global protein [44], which controls many different regulatory processes in the bacterial cell [55]. However, Taniguchi *et al*, [56], report that only a few hundred CsrA molecules are actually freely available in the bacterial cell (unbound CrsA proteins). Therefore, even small variations in the free CsrA concentration can have considerable effects. In the mutant strains CsrA2 and ΔLexA/CsrA2 the amount of free CsrA is directly affected, due to less CsrA proteins being able to bind to the respective binding sequence on the reporter plasmid. Furthermore, different CsrA sequestering elements have been reported, such as the sRNAs CsrB and CsrC [42,44] that are deleted in the CsrB and CsrBC mutants. Consequently, if CsrA abundance is increased (e.g. by reducing the amount of CsrA-sequestering sRNAs or by preventing CsrA binding to the SD sequence of the *cel* gene within the long mRNA), the noise of the entire ColicinE2-based operon (*cea* and *cel*) is reduced (Fig 4). We speculate this noise reduction to be due to transcriptional changes on a global protein interaction level. Here, different possible mechanisms can come into play. CsrA itself was shown to be a global negative regulator of transcription [57], that can cause transcription attenuation by mediating Rho-dependent termination [47] and furthermore regulates mRNA stability [47,57]. In addition, CsrA could act indirectly, by affecting the abundance of other global proteins. One such protein, whose expression is regulated by CsrA [58], is the protein Hfq that binds to mRNAs to initiate translation [59]. Hfq was also shown to be involved in the regulation of bacteriocin production [60]. Finally, strong *cel* translation that might be accompanied by a high density of ribosomes on the long mRNA could increase the stability of the long mRNA [61] and thus also affect *cea* gene expression noise.

The importance of various global factors for gene expression noise has been described previously [11,54,62]. With regard to noise generation in the ColicinE2-like system another global factor is important: the copy number of the plasmid carrying the ColicinE2 operon [26]. By reducing the plasmid copy number of our reporter plasmid from ~ 55 to ~ 13, we could show that indeed noise was strongly reduced, demonstrating the importance of the plasmid copy number, as a second global factor in our ColicinE2-based system for noise generation.

In summary, our data show that primarily post-transcriptional factors work together to control noise generation in the ColicinE2-based operon and indicate that these regulatory factors strongly affect noise in the native ColicinE2 toxin expression system, as well. Comparing the noise levels of the investigated mutant strains unexpectedly showed that the system closest to the native regulation (S strain) does not express the lowest noise. This leads us to the assumption that the native ColicinE2 system might have an optimized noise level that allows an increased variation of toxin expression and release throughout the heterogeneous population to increase the competitive success of the toxin-producing population. A similar effect was seen in a recent study, where the system of interest shows upregulation of population noise to enable bet-hedging in diverse environments [16].

As the plasmid-encoded system investigated in this study is controlled by chromosomally encoded global regulatory proteins such as LexA and CsrA, we furthermore hypothesize that modulation of gene expression noise by transcriptional and especially post-transcriptional factors might play an important role in comparable protein expression systems.

## Materials and methods

### Creation of bacterial strains used in this study

All strains used in this study are listed in S1 Table. All mutant strains are derivatives of the S strain described in Mader *et al*. [36] and Götz *et al*. [48]. Sequences of all mutant strains/plasmids have been verified.

The S strain, to which all mutant strain data are compared, carries the double reporter plasmid pMO3 that harbors the entire ColicinE2 operon, in which the genes *cea* and *cel* have been replaced by genes coding for the fluorescence proteins (FP) mVenus (YFP) and mCerulean (CFP), respectively (Fig 1A). Hence, this plasmid retains all regulatory sequences relevant for the binding of LexA to the SOS box of the ColicinE2 operon, and of CsrA to the Shine-Dalgarno sequence on the resulting long mRNA transcript. In contrast to the wild-type strain C_WT_ (Table 1, S1 Table), the S strain lacks the native pColE2-P9 plasmid and represents a mere reporter strain for ColicinE2 expression. Consequently, the S strain and its mutants are not able to produce or release the toxin via cell lysis.

The strain lacking the SOS box required for LexA binding (ΔLexA) was created as follows: We performed a PCR using the primer pair P1/P2 and the plasmid pMO3 (S2 Table) to create a DNA fragment without the SOS box sequence. The purified PCR product (QIAquick PCR Purification Kit, Qiagen, Hilden, Germany) containing a *KpnI* cutting site was cut with KpnI and DpnI and purified again in order to discard the original DNA Plasmid. The DNA fragment was then ligated to create the new plasmid present in ΔLexA (S1 Table). Due to the lack of the SOS box, this mutant constitutively expresses the ColicinE2-based operon (S8 Fig).

A further mutant ΔLexA/CsrA2 was constructed by performing site-directed mutagenesis on the plasmid in the ΔLexA mutant with the Quick ChangeII kit (Agilent Technologies, Santa Clara, USA). Using the primer pair P3/P4 (S2 Table), the CsrA binding site in Shine-Dalgarno sequence of the *cel/cfp* gene on the ΔLexA plasmid was switched from AC to TT. This leads to the new plasmid ΔLexA/CsrA2 (S1 Table) which is unable to form a second mRNA hairpin overlapping the Shine-Dalgarno sequence [41,63]. Consequently, CsrA binding is strongly reduced in this mutant, while transcription of the ColicinE2-based operon is constitutive.

To investigate the influence of the position of the fluorescent reporter proteins in the operon we created a mutant S_FLIP_, where the order of the two fluorescent reporter proteins in the operon was switched, leading to the replacement of *cea* by mCerulean and *cel* by mVenus. This mutant was created from the pMO3 plasmid in two steps. Step1: Using the primers P5/P6 for a PCR the YFP insert for *cel* position was duplicated. Accordingly, the primers P7/P8 were used in a PCR to create a vector pMO3 without CFP (at the *cel* position). After PCR purification (QIAquick PCR Purification Kit, Qiagen, Hilden, Germany) both vector and insert were cut with the restriction enzymes AgeI, BamHI and DpnI then ligated in a vector:insert ration of 1:3 and transformed into the XL1 strain. Step2: In order to replicate the DNA fragment for CFP at *cea* position the primer pair P9/P10 was used for PCR. The vector without *yfp* at *cea* position was created via PCR of the plasmid created in step1 with the pimers P11/P12. Both DNA fragments were then cut with the restriction enzymes EcoRI, SacI and DpnI. Further cloning steps were performed according to step 1, creating the final plasmid pMO11 that contains the entire ColicinE2 operon but with *cea* and *cel* exchanged by *cfp* and *yfp*, respectively.

All other mutants were created as described in Götz *et al*. [48] and detailed information on the mutations is included in S1 Table.

### Fluorescence time-lapse microscopy and data analysis

Bacterial cultivation, fluorescence time-lapse microscopy and data analysis have been performed as described earlier [48].

In detail, bacteria were grown overnight at 37°C in M63 minimal medium supplemented with 0.5% glycerol as a carbon source, and with 100 μg/ml ampicillin (Carl Roth, Germany) if required. Overnight cultures were diluted to an OD_600_ of 0.05 and grown to an OD_600_ of 0.2. Aliquots (50 μl) of these cultures were applied to poly-L-lysine (BIOCHROM, Berlin)-coated Ibidi μ-slides VI^0.4^ (Ibidi GmbH, Munich). These slides were then transferred to an inverse microscope, Axiovert 200M (Carl Zeiss, Germany) equipped with an Andor camera and a Zeiss EC Plan-Neofluar 100x/1.3 oil-immersion objective. A filter set with a beam splitter BS520, an excitation bandpass HC500/24 and an emission bandpass HC 542/27 was used for YFP detection. The HC filter set for CFP detection consisted of an emission filter 483/32, a beam splitter BS458 and an excitation filter 438/24. Micromanager, an open-source program (version 1.3), was used for image acquisition [64]. After the first image, the chamber was flushed with medium containing the appropriate concentration of mitomycin C (MitC, Carl Roth, Germany). For image analysis a background correction was performed using ImageJ [65] by calculating the modal gray from each single image. After that, stacks of the time traces were created and analyzed using the Cell Evaluator plug-in [66] for ImageJ. Only live cells lying within the bright-field image were considered. General data analysis was performed using IgorPRO 6.22 and 7.04, Adobe CS5 Software and Inkscape (Version 0.92.4).

We obtained the fluorescence intensity (FI) for YFP and CFP expression for each single cell every 15 min over a period of 300 min. As we did not observe significant photobleaching for the two fluorescent proteins, YFP and CFP, used in this study, both of which are known for their stable characteristics in protein folding, bleaching and fluorescence [67–69], we did not correct the obtained data for photobleaching.

All mutants except S_FLIP_ and S_REP2_ were measured with a fluorescence lamp intensity of 1% and 25ms exposure time for both channels. These values were chosen to ensure that no pixel saturation occurred for high FI values for any of the mutants studied. Due to their genetic changes the S_FLIP_ and S_REP2_ mutants had to be measured with a higher lamp intensity of 4% and 200ms exposure time in order to stay considerably above the detection limit. As changes in illumination have impact on both the fluorescence intensity and the width of the distribution (σ) this change should not have an impact on the CV. Consequently, only CV values of these two mutant strains can be compared to the values of the S strain.

The bacterial stress response was induced using medium containing high concentrations of Mitomycin C (0.1, 0.25 and 0.4 μg/ml) right after the first image was taken. To be able to quantify the noise of cells not or only weakly expressing the ColicinE2 operon from cells that are highly expressing the operon, a threshold level was set to distinguish expressers from non-expressers. As described earlier, a cell was defined to be in the ON state, if its FI level was five times higher than their basal fluorescence [36], with the exception of YFP expression in the ΔLexA and ΔLexA/CsrA2 mutants: here, all cells are expressing YFP constitutively. Consequently, all cells of these two mutants are permanently in the YFP ON state (S8 Fig). As a standard measure of noise in gene expression [23], we quantified the coefficient of variation (CV) for the obtained fluorescence intensities (FI), being the standard deviation σ divided by the mean μ. We studied noise generation at high externals stress levels, consequently, the cells do no longer divide. Furthermore, the maximal amount of cells have finished switching at the end of the measurement and there is no significant difference in the cells overall response between the different MitC concentrations. Thus, we calculated the mean FI, σ and CV with 95% confidence interval as error bars for the different datasets from all three stress levels, if not indicated otherwise. For each MitC concentration at least 2 separate experiments have been performed and a minimum of 93 cells has been analyzed. In S3 Table detailed information on the measured MitC concentrations for each strain/mutant, the resulting number of replicates N and the number of considered cells X for data analysis can be found. Further information on the exact number of cells analyzed for YFP or CFP expression is given in the S1 Data file.

### Significance analysis

Significance analysis was performed using statistical programming language R and R Studio and the available ‘stats’ library. First, the distributions were tested for normality using the shapiro.test function. Depending on the results, significance analysis was performed using the t.test function for normal distributions and the wilcoxon.test function for non-normal distributions. The significance is depicted in figures as follows: * p<0.05; ** p<0.01; *** p<0.001 and n.s. (not significant) for p>0.05. More detailed information of the significance values is given in the S1 Data file.

## Supporting information

S1 Table Bacterial strains used in this study. (PDF)

S2 Table Primers used in this study. These primers were used for construction of the mutant strains created in this study described in Materials and methods.
(PDF)

S3 Table Measurement overview. Detailed information on collected and analyzed data for all bacterial strains and mutants used in this study. This includes a summary of the MitC concentrations measured for a particular strain/mutant, the resulting number of replicates N and the total number of cells X considered for each strain/mutant in data analysis. Information on the exact cell numbers for ON/OFF states, YFP and CFP expression is given in the S1 Data file.
(PDF)

S1 Fig Fluorescence intensity data for the expression of YFP and CFP in the S strain. A,B) Mean Fluorescence Intensity (FI) over time for all cells. Squares: 0.1 μg/ml MitC, triangles: 0.25 μg/ml MitC, circles: 0.4 μg/ml. (C-H) FI histograms for OFF state (C,D), ON state (E,F) and all cells (G,H) are shown. The plots for expression of YFP (A,C,E,G) and CFP (B,D,F,H) are plotted in yellow and blue respectively. In all histograms the MitC concentration is given by bar color from light to dark with increasing MitC levels. The subfigures for mean FI and σ are given in each plot for the subpopulations of all MitC concentration (0.10 μg/ml, 0.25 μg/ml, 0.40 μg/ml MitC). Detailed information on experimental replicates N and analyzed cell numbers X can be found in S3 Tableand the S1 Data file.
(TIF)

S2 Fig Noise of S strain in the absence of MitC. A,B) Coefficient of variation (CV) over time of all cells not expressing the ColicinE2 operon. The low fraction of cells switching to the ON-state in the absence of MitC has not been included in these data, to allow a direct comparison of the OFF-state values of S strain in the presence of MitC. Open squares: no MitC, filled triangles: S strain cells OFF-state in the presence of MitC, averaged over all cells X. A) YFP expression (yellow), B) CFP expression (blue), C) Basal noise for YFP and CFP expression in the absence of MitC. The basal noise was quantified as noise of the respective populations at t = 45 min. The error bar denotes the 95% confidence interval around the mean of all measurements. Transparent areas in yellow (YFP) and blue (CFP) indicate the basal noise values for S strain in the presence of MitC (*cea* (*yfp*), *cel* (*cfp*)). Number of replicates N for each bar in (C) is 2, with 213 considered cells in total. Detailed information on analyzed cell numbers can be found in the S1 Data file.
(TIF)

S3 Fig Standard deviation and mean of fluorescence intensity of cells in the ON state for YFP expression (S strain). A) Mean standard deviation (σ) and mean fluorescence intensity (FI) of cells in the ON state over time. B) Mean σ and mean FI of cells in the ON state in comparison to cells in the OFF state. Values represent ratios of σ: mean FI. The error bar denotes the 95% confidence interval around the mean of all measurements. Number of replicates N for each bar shown in (B) is 8, with in total 310 considered cells. Detailed information on analyzed cell numbers can be found in the S1 Data file.
(TIF)

S4 Fig Gene expression noise and FI histograms of the SFLIP mutant with switched FPs compared to the S strain. A) Noise in *cea* (*cfp*) expression is given in blue, noise in *cel* (*yfp*) expression is given in yellow. Transparent areas yellow (YFP) and in blue (CFP) indicate the noise values for the S strain (*cea* (*yfp*), *cel* (*cfp*), Materials and methods, S1 Table) Basal noise of cells in the OFF state (left). Noise of cells in the ON state (middle). Population noise (right). Significance levels of the corresponding S_FLIP_ distributions: n.s. no significant difference; **: p<0.01. B/C) Histograms of fluorescence intensity distribution of the entire population FI at 270min for B) *cea* (*cfp*) and C) *cel* (*yfp*) of the S_FLIP_ mutant for all measurements. The error bar denotes the 95% confidence interval around the mean of all measurements. Number of replicates N for each bar shown in (A) is 3, with 143 considered cells in total. Detailed information on analyzed cell numbers can be found in the S1 Data file.
(TIF)

S5 Fig Noise of cells in the OFF (basal noise) and in the ON state in various mutant strains (Materials and methods, Table 1 and S1 Table). Coefficient of variation (CV) of YFP gene expression is shown in yellow, of CFP expression in blue. A,B) Noise of genes in bacterial cells in the OFF state. C,D) noise of genes in bacterial cells in the ON state. Background color indicates genetic changes in transcriptional (T, light grey) or post-transcriptional regulation (PT, medium grey), or both (T and PT, dark grey). Transparent colored regions (YFP, yellow and CFP, blue) indicate the CV values (with the corresponding error) of the S strain for better comparability between data sets. Significance levels are set as n.s. no significant difference; *: p<0.05; **: p<0.01; ***: p<0.001 and represent the comparison to the S strain. The error bar denotes the 95% confidence interval around the mean of all measurements. Number of replicates N for each strain is S: 8, ΔLexA: 9, LexA1: 11, LexA2: 10, CsrA1: 9, CsrB: 6, CsrBC: 7, CsrA2: 7, ΔLexA/CsrA2: 7. Total number of considered cells X: S: 310, ΔLexA: 301, LexA1: 431, LexA2: 382, CsrA1: 434, CsrB: 314, CsrBC: 312, CsrA2: 348, ΔLexA/CsrA2: 247. Detailed information on analyzed cell numbers (ON/OFF state, YFP/CFP) can be found in the S1 Data file.
(TIF)

S6 Fig Mean FI and σ of the entire population (corresponding to population noise at 270min) in various mutant strains (Materials and methods, Table 1 and S1 Table). A) Mean FI and B) mean σ of YFP gene expression are shown in yellow. C) Mean FI and D) mean σ of CFP expression are shown in blue. Background color indicates genetic changes in transcriptional (T, light grey) or post-transcriptional regulation (PT, medium grey), or both (T and PT, dark grey). Transparent colored regions (YFP, yellow and CFP, blue) indicate the corresponding values (with error) of the S strain for better comparability between data sets. Values highlighted in white are the FI and σ values for the corresponding bars as they were much higher than those of the other mutants and axis was cut for better visibility. The error bar denotes the 95% confidence interval around the mean of all measurements. Number of replicates N for each strain is S: 8, ΔLexA: 9, LexA1: 11, LexA2: 10, CsrA1: 9, CsrB: 6, CsrBC: 7, CsrA2: 7, ΔLexA/CsrA2: 7. Number of considered cells X: S: 310, ΔLexA: 301, LexA1: 431, LexA2: 382, CsrA1: 434, CsrB: 314, CsrBC: 312, CsrA2: 348, ΔLexA/CsrA2: 247. Detailed information on analyzed cell numbers can be found in S3 Table.
(TIF)

S7 Fig Histograms for YFP and CFP fluorescence intensities for all mutants with CVs significantly different from the S strain. A,C,E,G) YFP expression and B,D,F,H) CFP expression histograms for all measured cells at three MitC concentrations are depicted in yellow and blue respectively. Histograms are corresponding to the population noise state of all cells at 270min of the measurement.
(TIF)

S8 Fig Constitutive expression of the ColicinE2-based operon in the mutant strain Δ LexA. A) Single traces of 50 cells expressing YFP over time (grey). B) Fraction of cells expressing YFP and CFP in Δ LexA. Please note that the long mRNA including the *cfp* gene (Fig 1) is less frequent. Furthermore, the *cfp* gene underlies additional post-transcriptional regulation by CsrA. Hence, the fraction of cells expressing *cfp* is not 100%. The error of the cumulative fraction of cells in the ON state is given by the standard error of the mean (SEM) with the error bar.
(TIF)

S9 Fig Gene expression noise and FI histograms of the SREP2 strain with reduced plasmid copy number (~ 13copies). A) Noise in YFP expression is given in yellow, noise in CFP expression is given in blue. Transparent areas yellow (YFP) and in blue (CFP) indicate the noise values for the S strain with high plasmid copy number (~ 55 copies, Materials and methods, S1 Table) Basal noise of cells in the OFF state (left). Noise of cells in the ON state (middle). Population noise (right). Significance levels of the corresponding S_REP2_ distribution compared to the S strain: n.s. no significant difference; *: p<0.05; **: p<0.01; ***: p<0.001. B/C) Histograms of fluorescence intensity distribution of population FI at 270min on B) *cea* (*yfp*) and C) *cel* (*cfp*) of the S_REP2_ strain for all measurements. The error bar denotes the 95% confidence interval around the mean of all measurements. Number of replicates N for each bar shown in (A) is 3, with 93 considered cells in total. Detailed information on analyzed cell numbers can be found in the S1 Data file.
(TIF)

S1 Data This excel file includes more detailed information for data presented in the figures and significance analysis. (XLSX)

## References

[1] Elowitz MB, Levine AJ, Siggia ED, Swain PS. Stochastic gene expression in a single cell. Science (80-). 2002;297: 1183–1187. 10.1126/science.1070919 12183631

[2] Kærn M, Elston TC, Blake WJ, Collins JJ. Stochasticity in gene expression: From theories to phenotypes. Nat Rev Genet. 2005;6: 451–464. 10.1038/nrg1615 15883588

[3] McAdams HH, Arkin A. Stochastic mechanisms in gene expression. Proc Natl Acad Sci. 1997;94: 814–819. 10.1073/pnas.94.3.814 9023339 PMC19596

[4] Losick R, Desplan C. Stochasticity and Cell Fate. Science (80-). 2008;320: 65–68. 10.1126/science.1147888 18388284 PMC2605794

[5] Ferguson ML, Le Coq D, Jules M, Aymerich S, Radulescu O, Declerck N, et al Reconciling molecular regulatory mechanisms with noise patterns of bacterial metabolic promoters in induced and repressed states. Proc Natl Acad Sci. 2012;109: 155–160. 10.1073/pnas.1110541108 22190493 PMC3252923

[6] Sanchez A, Choubey S, Kondev J. Regulation of Noise in Gene Expression. Annu Rev Biophys. 2013;42: 469–491. 10.1146/annurev-biophys-083012-130401 23527780

[7] Ozbudak E, Thattai M, Kurtser I, Grossman A, van Oudenaarden A. Regulation of noise in the expression of a single gene. Nat Genet. 2002; 10.1038/ng869 11967532

[8] Murphy KF, Adams RM, Wang X, Balázsi G, Collins JJ. Tuning and controlling gene expression noise in synthetic gene networks. Nucleic Acids Res. 2010;38: 2712–2726. 10.1093/nar/gkq091 20211838 PMC2860118

[9] Hansen MMK, Weinberger LS. Post-Transcriptional Noise Control. BioEssays. 2019;41: 1–10. 10.1002/bies.201900130 31222776 PMC6637019

[10] Kleijn IT, Krah LHJ, Hermsen R. Noise propagation in an integrated model of bacterial gene expression and growth. PLoS Comput Biol. 2018;14: 1–18. 10.1371/journal.pcbi.1006386 30289879 PMC6192656

[11] Pedraza JM, van Oudenaarden A. Noise propagation in gene networks. Science (80-). 2005;307: 1–12. 10.1126/science.1109090 15790857

[12] Newman JRS, Ghaemmaghami S, Ihmels J, Breslow DK, Noble M, DeRisi JL, et al Single-cell proteomic analysis of S. cerevisiae reveals the architecture of biological noise. Nature. 2006;441: 840–846. 10.1038/nature04785 16699522

[13] Blake WJ, Kærn M, Cantor CR, Collins JJ. Noise in eukaryotic gene expression. 2003;249: 247–249. 10.1038/nature01546 12687005

[14] Mundt M, Anders A, Murray SM, Sourjik V. A System for Gene Expression Noise Control in Yeast. ACS Synth Biol. 2018;7: 2618–2626. 10.1021/acssynbio.8b00279 30354070 PMC6243393

[15] Silander OK, Nikolic N, Zaslaver A, Bren A, Kikoin I, Alon U, et al A Genome-Wide Analysis of Promoter-Mediated Phenotypic Noise in Escherichia coli Olin. Plos Gene. 2012;8 10.1371/journal.pgen.1002443 22275871 PMC3261926

[16] Carey JN, Goulian M. A bacterial signaling system regulates noise to enable bet hedging. Curr Genet. 2018; 1–6. 10.1007/s00294-018-0856-2 29947971 PMC6291380

[17] Ackermann M. A functional perspective on phenotypic heterogeneity in microorganisms. Nat Rev Microbiol. 2015;13: 497–508. 10.1038/nrmicro3491 26145732

[18] Colin R, Rosazza C, Vaknin A, Sourjik V. Multiple sources of slow activity fluctuations in a bacterial chemosensory network. Elife. 2017;6: 1–32. 10.7554/eLife.26796 29231168 PMC5809148

[19] Engl C. Noise in bacterial gene expression. Biochem Soc Trans. 2018;47: 209–217. 10.1042/BST20180500 30578346

[20] Wang Z, Zhang J. Impact of gene expression noise on organismal fitness and the efficacy of natural selection. Proc Natl Acad Sci. 2011;108: E67–E76. 10.1073/pnas.1100059108 21464323 PMC3080991

[21] Kussell E. Phenotypic Diversity, Population Growth, and Information in Fluctuating Environments. Science (80-). 2005;309: 2075–2078. 10.1126/science.1114383 16123265

[22] Raj A, van Oudenaarden A. Nature, Nurture, or Chance: Stochastic Gene Expression and Its Consequences. Cell. 2008;135: 216–226. 10.1016/j.cell.2008.09.050 18957198 PMC3118044

[23] Raser JM, O’Shea EK. Noise in Gene Expression: Origins, Consequences, and Control. Science (80-). 2005;309: 2010 LP– 2013. Available: http://science.sciencemag.org/content/309/5743/2010.abstract 16179466 10.1126/science.1105891 PMC1360161

[24] Li GW, Xie XS. Central dogma at the single-molecule level in living cells. Nature. 2011;475: 308–315. 10.1038/nature10315 21776076 PMC3600414

[25] Balázsi G, Van Oudenaarden A, Collins JJ. Cellular decision making and biological noise: From microbes to mammals. Cell. 2011;144: 910–925. 10.1016/j.cell.2011.01.030 21414483 PMC3068611

[26] Cascales E, Buchanan SK, Duche D, Kleanthous C, Lloubes R, Postle K, et al Colicin Biology. Microbiol Mol Biol Rev. 2007;71: 158–229. 10.1128/MMBR.00036-06 17347522 PMC1847374

[27] Wu PJ, Shannon K, Phillips I. Mechanisms of hyperproduction of TEM-1 β-lactamase by clinical isolates of escherichia coli. J Antimicrob Chemother. 1995;36: 927–939. 10.1093/jac/36.6.927 8821592

[28] Millan AS, Escudero JA, Gifford DR, Mazel D, Maclean RC. Multicopy plasmids potentiate the evolution of antibiotic resistance in bacteria. Nat Ecol Evol. 2016; 10.1038/s41559-016-0010 28812563

[29] Kerr B, Riley MA, Feldman MW, Bohannan BJM. Local dispersal promotes biodiversity in a real-life game of rock-paper-scissors. Nature. 2002;418: 171–174. 10.1038/nature00823 12110887

[30] Kelsic ED, Zhao J, Vetsigian K, Kishony R. Counteraction of antibiotic production and degradation stabilizes microbial communities. Nature. 2015;521: 516–519. 10.1038/nature14485 25992546 PMC4551410

[31] Kirkup BC, Riley MA. Antibiotic-mediated antagonism leads to a bacterial game of rock–paper–scissors in vivo. Nature. 2004;428: 694–696. 10.1038/nature02429 15042087

[32] Lechner M, Schwarz M, Opitz M, Frey E. Hierarchical Post-transcriptional Regulation of Colicin E2 Expression in Escherichia coli. PLoS Comput Biol. 2016;12: 1–20. 10.1371/journal.pcbi.1005243 27977665 PMC5157957

[33] Reichenbach T, Mobilia M, Frey E. Mobility promotes and jeopardizes biodiversity in rock-paper-scissors games. Nature. 2007;448: 1046–1049. 10.1038/nature06095 17728757

[34] von Bronk B, Schaffer SA, Götz A, Opitz M. Effects of stochasticity and division of labor in toxin production on two-strain bacterial competition in Escherichia coli. PLoS Biol. 2017;15: 1–25. 10.1371/journal.pbio.2001457 28459803 PMC5411026

[35] von Bronk B, Götz A, Opitz M. Locality of interactions in three-strain bacterial competition. Phyisical Biol. 2019;16. 10.1088/1478-3975/aae671 30376449

[36] Mader A, von Bronk B, Ewald B, Kesel S, Schnetz K, Frey E, et al Amount of Colicin Release in Escherichia coli Is Regulated by Lysis Gene Expression of the Colicin E2 Operon. PLoS One. 2015;10: e0119124 10.1371/journal.pone.0119124 25751274 PMC4353708

[37] Mrak P, Podlesek Z, Van Putten JPM, Žgur-Bertok D. Heterogeneity in expression of the Escherichia coli colicin K activity gene cka is controlled by the SOS system and stochastic factors. Mol Genet Genomics. 2007;277: 391–401. 10.1007/s00438-006-0185-x 17216493

[38] Kamenšek S, Podlesek Z, Gillor O, Žgur-Bertok D. Genes regulated by the Escherichia coli SOS repressor LexA exhibit heterogenous expression. BMC Microbiol. 2010;10: 283 10.1186/1471-2180-10-283 21070632 PMC2994835

[39] Ozeki H, Stocker B, De Margerie H. Production of colicine by single bacteria. Nature. 1959;184. 10.1038/184337a0 14429601

[40] Riley M a., Wertz JE. Bacteriocins: Evolution, Ecology, and Application. Annu Rev Microbiol. 2002;56: 117–137. 10.1146/annurev.micro.56.012302.161024 12142491

[41] Yang TY, Sung YM, Lei GS, Romeo T, Chak KF. Posttranscriptional repression of the cel gene of the ColE7 operon by the RNA-binding protein CsrA of Escherichia coli. Nucleic Acids Res. 2010;38: 3936–3951. 10.1093/nar/gkq177 20378712 PMC2896534

[42] Weilbacher T, Suzuki K, Dubey AK, Wang X, Gudapaty S, Morozov I, et al A novel sRNA component of the carbon storage regulatory system of Escherichia coli. Mol Microbiol. 2003;48: 657–670. 10.1046/j.1365-2958.2003.03459.x 12694612

[43] Suzuki K, Babitzke P, Kushner SR, Romeo T. Identification of a novel regulatory protein (CsrD) that targets the global regulatory RNAs CsrB and CsrC for degradation by RNase E. Genes Dev. 2006;20: 2605–2617. 10.1101/gad.1461606 16980588 PMC1578682

[44] Gudapaty S, Suzuki K, Wang X, Romeo T, Wang XIN, Babitzke P. Regulatory Interactions of Csr Components: the RNA Binding Protein CsrA Activates *csrB* Transcription in *Escherichia coli*. J Bacteriol. 2001;183: 6017–6027. 10.1128/JB.183.20.6017-6027.2001 11567002 PMC99681

[45] Babitzke P, Romeo T. CsrB sRNA family: sequestration of RNA-binding regulatory proteins. Curr Opin Microbiol. 2007;10: 156–163. 10.1016/j.mib.2007.03.007 17383221

[46] Vakulskas CA, Leng Y, Abe H, Amaki T, Okayama A, Babitzke P, et al Antagonistic control of the turnover pathway for the global regulatory sRNA CsrB by the CsrA and CsrD proteins. Nucleic Acids Res. 2016;44: 7896–7910. 10.1093/nar/gkw484 27235416 PMC5027483

[47] Romeo T, Babitzke P. Global Regulation by CsrA and Its RNA Antagonists. Microbiol Spectr. 2018;6: 1–14. 10.1128/microbiolspec.RWR-0009-2017.Correspondence PMC5868435 29573256

[48] Götz A, Lechner M, Mader A, von Bronk B, Frey E, Opitz M. CsrA and its regulators control the time-point of ColicinE2 release in Escherichia coli. Sci Rep. 2018;8: 6537 10.1038/s41598-018-24699-z 29695793 PMC5916893

[49] Shimoni Y, Altuvia S, Margalit H, Biham O. Stochastic Analysis of the SOS Response in Escherichia coli. PLoS One. 2009;4: e5363 Available: 10.1371/journal.pone.0005363 19424504 PMC2675100

[50] Vimberg V, Tats A, Remm M, Tenson T. Translation initiation region sequence preferences in Escherichia coli. BMC Mol Biol. 2007;8: 100 10.1186/1471-2199-8-100 17973990 PMC2176067

[51] Cole ST, Saint-Joanis B PA. Molecular characterisation of the colicin E2 operon and identification of its products. Mol Gen Genet. 1985;24: 198(3):465–72. 10.1007/bf00332940 3892228

[52] Silva JPN, Lopes SV, Grilo DJ, Hensel Z. Plasmids for Independently Tunable, Low-Noise Expression of Two Genes. mSphere. 2019; 1–9. 10.1128/mSphere.00340-19. Editor PMC6541738 31142623

[53] Dacheux E, Malys N, Xiang M, Ramachandran V, Mendes P, McCarthy JEG. Translation initiation events on structured eukaryotic mRNAs generate gene expression noise. Nucleic Acids Res. 2017;45: 6981–6992. 10.1093/nar/gkx430 28521011 PMC5499741

[54] Bar-Even A, Paulsson J, Maheshri N, Carmi M, O’Shea E, Pilpel Y, et al Noise in protein expression scales with natural protein abundance. Nat Genet. 2006;38: 636–643. 10.1038/ng1807 16715097

[55] Romeo T. Global regulation by the small RNA-binding protein CsrA and the non- coding RNA molecule CsrB. Mol Microbiol. 1998;29: 1321–1330. 10.1046/j.1365-2958.1998.01021.x 9781871

[56] Taniguchi Y, Choi PJ, Li G, Chen H, Babu M, Hearn J, et al Quantifying E. coli proteome and transcriptome with single-molecule sensitivity in single cells. Science (80-). 2010;329: 533–539. 10.1126/science.1188308 20671182 PMC2922915

[57] Esquerré T, Bouvier M, Turlan C, Carpousis AJ, Girbal L, Cocaign-Bousquet M. The Csr system regulates genome-wide mRNA stability and transcription and thus gene expression in Escherichia coli. Sci Rep. 2016;6: 25057 10.1038/srep25057 27112822 PMC4844966

[58] Baker CS, Eöry L a., Yakhnin H, Mercante g, Romeo T, Babitzke P. CsrA inhibits translation initiation of Escherichia coli hfq by binding to a single site overlapping the Shine-Dalgarno sequence. J Bacteriol. 2007;189: 5472–5481. 10.1128/JB.00529-07 17526692 PMC1951803

[59] Vassilieva IM, Garber MB. The regulatory role of the Hfq protein in bacterial cells. Mol Biol. 2002;36: 785–791. 10.1023/A:1021621623503 12500533

[60] Valentin-Hansen P. Structure, function and RNA binding mechanisms of the prokaryotic Sm-like protein hfq Regulatory RNAs in Prokaryotes. Springer; 2012 pp. 147–162.

[61] Edri S, Tuller T. Quantifying the effect of ribosomal density on mRNA stability. PLoS One. 2014;9 10.1371/journal.pone.0102308 25020060 PMC4096589

[62] Jones DL, Brewster RC, Phillips R. Promoter architecture dictates cell-to-cell variability in gene expression. Science (80-). 2014;346: 1533–1536. 10.1126/science.1255301 25525251 PMC4388425

[63] Hol FJH, Voges MJ, Dekker C, Keymer JE. Nutrient-responsive regulation determines biodiversity in a colicin-mediated bacterial community. BMC Biol. 2014; 1–14. 10.1186/1741-7007-12-1 25159553 PMC4161892

[64] Edelstein A, Amodaj N, Hoover K, Vale R, Stuurman N. Computer Control of Microscopes Using MicroManager. Curr Protoc Mol Biol. 2010; 10.1002/0471142727.mb1420s92 20890901 PMC3065365

[65] Rasband WS (USNI of H. ImageJ [Internet]. Available: http://imagej.nih.gov/ij/

[66] Youssef S, Gude S, Radler JO. Automated tracking in live-cell time-lapse movies. Integr Biol. 2011;3: 1095–1101. 10.1039/C1IB00035G 21959912

[67] Kremers G, Goedhart J, Munster EB Van, Gadella TWJ. Cyan and Yellow Super Fluorescent Proteins with Improved Brightness, Protein Folding, and FRET F ö rster Radius Cyan and Yellow Super Fluorescent Proteins with Improved Brightness, Protein Folding, and FRET Fo. Biochemistry. 2006; 6570–6580. 10.1021/bi0516273 16716067

[68] Kremers GJ, Goedhart J, Van Den Heuvel DJ, Gerritsen HC, Gadella TWJ. Improved green and blue fluorescent proteins for expression in bacteria and mammalian cells. Biochemistry. 2007;46: 3775–3783. 10.1021/bi0622874 17323929

[69] Markwardt ML, Kremers GJ, Kraft C a, Ray K, Cranfill PJC, Wilson K a., et al An improved cerulean fluorescent protein with enhanced brightness and reduced reversible photoswitching. PLoS One. 2011;6 10.1371/journal.pone.0017896 21479270 PMC3066204

