# Supplementary material for: Gene expression noise in a complex artificial toxin expression system
Source: PLoS One. 2020 Jan 21;15(1):e0227249. doi: 10.1371/journal.pone.0227249 (PMC6974158; doi:10.1371/journal.pone.0227249)
Supplement: S1 Table — (PDF) [file pone.0227249.s001.pdf]

Bacterial strain	Strain description	Genetic modification/information
C_{WT} (EMO3-C) [1]	BZB 1011 E2C	Carries native toxin producing plasmid pColE2-P9
S (EMO3-S) [1]	BZB 1011 pMO3	Carries YFP - CFP double reporter plasmid pMO3
LexA1 [2]	BZB 1011 pMO4	LexA binding sequence altered on pMO3, resulting in pMO4
LexA2 [2]	BZB 1011 pMO5	LexA binding sequence altered on pMO3, resulting in pMO5
Δ LexA<br>This study	BZB 1011 pDelLexA	SOS box sequence deleted on pMO3, resulting in pDelLexA
CsrA1 [2]	BZB 1011 pMO6	CsrA binding sequence on pMO3 altered to achieve stronger CsrA binding, resulting in pMO6
CsrA2 [2]	BZB 1011 pMO7	CsrA binding sequence on pMO3 altered, resulting in pMO7
CsrB [2] (EMO3::CsrB)	BZB 1011 CsrB::Kan pMO3	CsrB::Kan, in-frame replacement of CsrB by a kanamycin resistance
CsrBC [2] (EMO3::CsrBC)	BZB 1011 CsrB::Cam CsrC::Kan pMO3	CsrC::Kan, CsrB::Cam, in-frame replacement of CsrC by a kanamycin resistance and of CsrB by a chloramphenicol resistance cassette
Δ LexA/CsrA2<br>This study	BZB 1011 pDelLexA-CsrA2	SOS box sequence deleted on pMO3, resulting in pDelLexA, CsrA binding sequence on pMO3 altered, resulting in pMO7
S_{REP2} [2]	BZB 1011 pMO8	Origin of replication on pMO3 has been changed to p15A to achieve a lower copy number of 13 copies per cell -> pMO8
S_{FLIP}	BZB 1011 pMO11	Carries CFP - YFP double reporter plasmid, where the fluorescent proteins are switched compared to pMO3
