# Supplementary material for: Gene expression noise in a complex artificial toxin expression system
Source: PLoS One. 2020 Jan 21;15(1):e0227249. doi: 10.1371/journal.pone.0227249 (PMC6974158; doi:10.1371/journal.pone.0227249)
Supplement: S2 Table — These primers were used for construction of the mutant strains created in this study described in Materials and methods. (PDF) [file pone.0227249.s002.pdf]

Name	Sequence	Purpose
P1	5´- GCTCGGTACCATTAATCATGTAATTAATTG- 3´	Δ LexA [fwd] cloning
P2	5´- ATATGGTACCCAAAAAGTACCCGTCAT - 3´	Δ LexA [rev] cloning
P3	5´- GCATTCTTTCACAACAAGGATGTGTTATGAAAAAAATAACCGG-3´	Δ LexA/CsrA2 [fwd] cloning
P4	5´- CCGGTTATTTTTTTCATAACACATCCTTGTTGTGAAAGAATGC- 3´	Δ LexA/CsrA2 [rev] cloning
P5	5´- ATTTACCGGTATGAGCAAGGGCGAG- 3´	FLIP[fwd] cloning *yfp->cel*
P6	5´- ATATGGATCCTTACTTGTACAGCTCGTC- 3´	FLIP[rev] cloning *yfp->cel*
P7	5´- TTATTACCGGTTATTTTTTTCATAACG- 3´	FLIP[fwd] cloning pMO3 without *cfp*
P8	5´- TTATTGGATCCATCCTCTTTGAC- 3´	FLIP[rev] cloning pMO3 without *cfp*
P9	5´- ATATGAATTCATGGTCAGCAAGG- 3´	FLIP[fwd]cloning *cfp->cea*
P10	5´- ATATGAGCTCTTACTTGTACAGCTC- 3´	FLIP[rev] cloning *cfp->cea*
P11	5´- TTATAGAATTCCCTCTTTTAAGC- 3´	FLIP[fwd] cloning vector without *yfp* at *cea* position
P12	5´- TTATTGAGCTCCATCATGATAAAC- 3´	FLIP[rev] cloning vector without *yfp* at *cea* position
