# Supplementary material for: Gene expression noise in a complex artificial toxin expression system
Source: PLoS One. 2020 Jan 21;15(1):e0227249. doi: 10.1371/journal.pone.0227249 (PMC6974158; doi:10.1371/journal.pone.0227249)
Supplement: S3 Table — Detailed information on collected and analyzed data for all bacterial strains and mutants used in this study. This includes a summary of the MitC concentrations measured for a particular strain/mutant, the resulting number of replicates N and the total number of cells X considered for each strain/mutant in data analysis. Information on the exact cell numbers for ON/OFF states, YFP and CFP expression is given in the S1 Data file. (PDF) [file pone.0227249.s003.pdf]

Strain	S	ΔLexA	LexA1	LexA2	CsrA1	CsrB	CsrBC	CsrA2	ΔLexA/ CsrA2	S_{FLIP}	S_{REP2}
**MitC Concentrations Measured [µg/ml]**	0.10; 0.25; 0.40	0.10; 0.25; 0.40	0.10; 0.25; 0.40	0.10; 0.25; 0.40	0.10; 0.25; 0.40	0.10; 0.25; 0.40	0.10; 0.25; 0.40	0.10; 0.25; 0.40	0.10; 0.25; 0.40	0.25	0.10
**Replicates N**	8	9	11	10	9	6	7	7	7	3	3
**Total Number of Cells X**	310	301	431	382	434	314	312	348	247	143	93
