# Supplementary figures and images for: Gene expression noise in a complex artificial toxin expression system
Source: PLoS One. 2020 Jan 21;15(1):e0227249. doi: 10.1371/journal.pone.0227249 (PMC6974158; doi:10.1371/journal.pone.0227249)

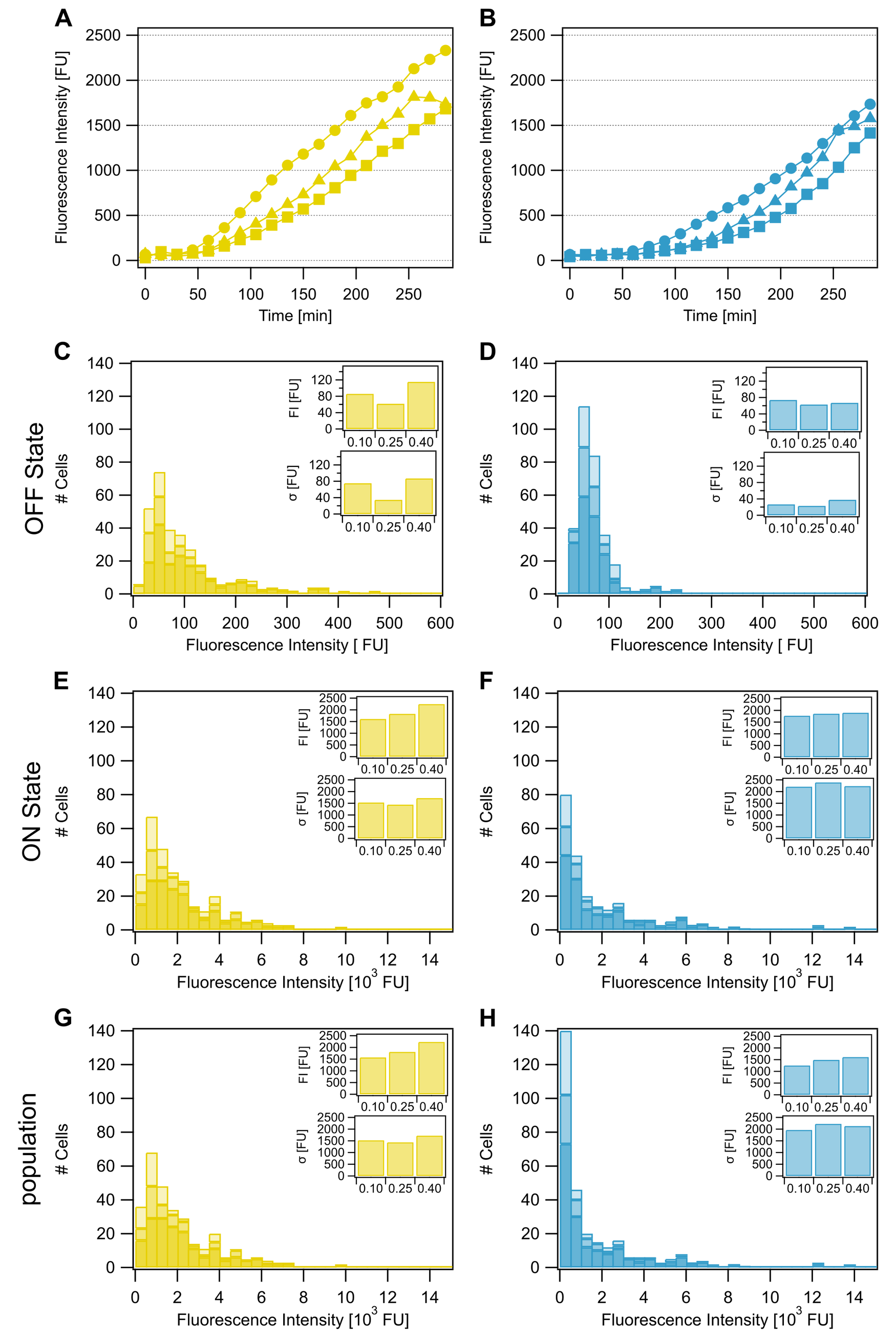

Supplement: S1 Fig — A,B) Mean Fluorescence Intensity (FI) over time for all cells. Squares: 0.1 μg/ml MitC, triangles: 0.25 μg/ml MitC, circles: 0.4 μg/ml. (C-H) FI histograms for OFF state (C,D), ON state (E,F) and all cells (G,H) are shown. The plots for expression of YFP (A,C,E,G) and CFP (B,D,F,H) are plotted in yellow and blue respectively. In all histograms the MitC concentration is given by bar color from light to dark with increasing MitC levels. The subfigures for mean FI and σ are given in each plot for the subpopulations of all MitC concentration (0.10 μg/ml, 0.25 μg/ml, 0.40 μg/ml MitC). Detailed information on experimental replicates N and analyzed cell numbers X can be found in S3 Tableand the S1 Data file. (TIF) [file pone.0227249.s004.tif]

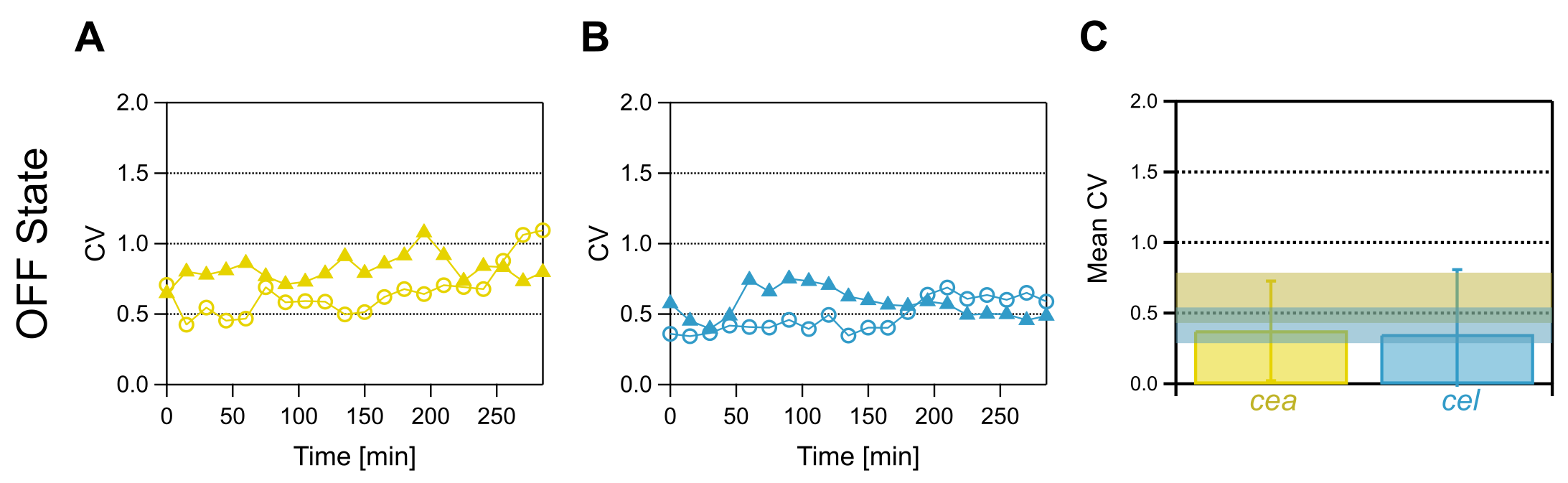

Supplement: S2 Fig — A,B) Coefficient of variation (CV) over time of all cells not expressing the ColicinE2 operon. The low fraction of cells switching to the ON-state in the absence of MitC has not been included in these data, to allow a direct comparison of the OFF-state values of S strain in the presence of MitC. Open squares: no MitC, filled triangles: S strain cells OFF-state in the presence of MitC, averaged over all cells X. A) YFP expression (yellow), B) CFP expression (blue), C) Basal noise for YFP and CFP expression in the absence of MitC. The basal noise was quantified as noise of the respective populations at t = 45 min. The error bar denotes the 95% confidence interval around the mean of all measurements. Transparent areas in yellow (YFP) and blue (CFP) indicate the basal noise values for S strain in the presence of MitC (cea (yfp), cel (cfp)). Number of replicates N for each bar in (C) is 2, with 213 considered cells in total. Detailed information on analyzed cell numbers can be found in the S1 Data file. (TIF) [file pone.0227249.s005.tif]

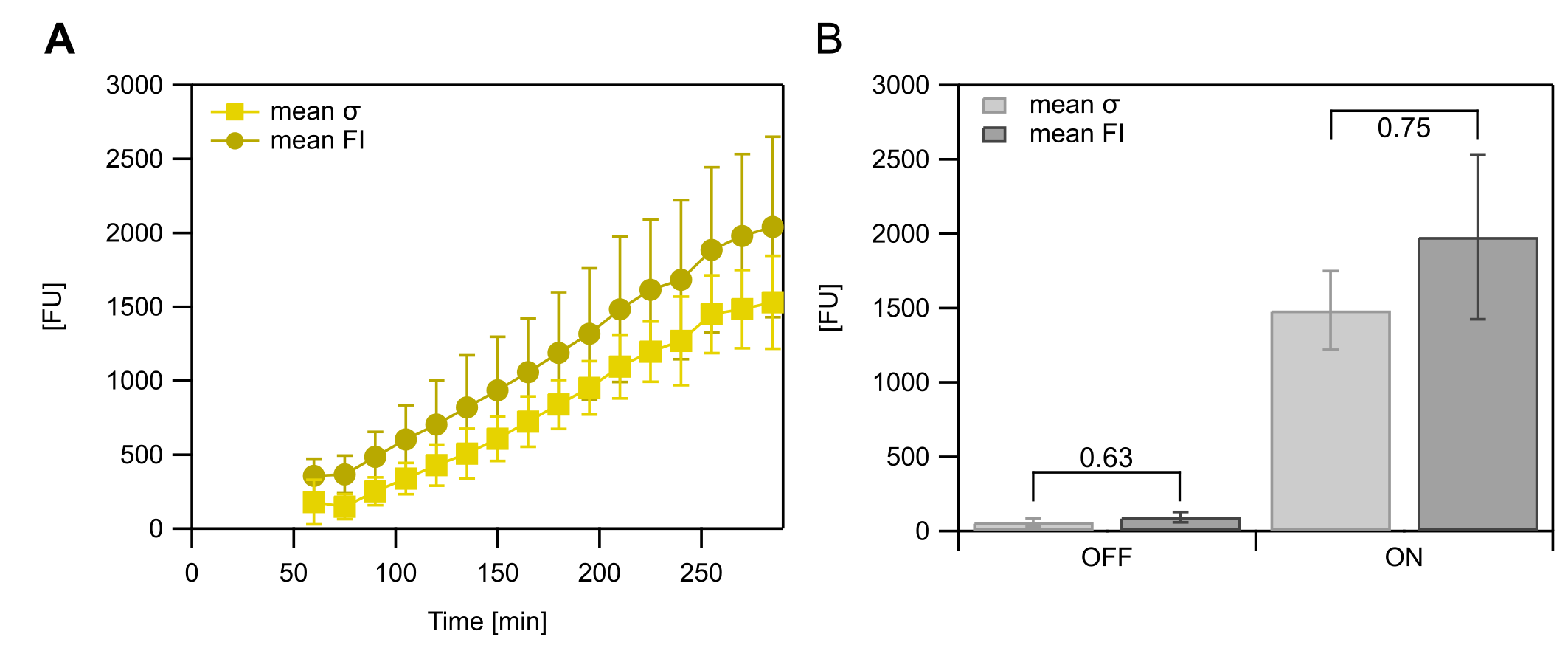

Supplement: S3 Fig — A) Mean standard deviation (σ) and mean fluorescence intensity (FI) of cells in the ON state over time. B) Mean σ and mean FI of cells in the ON state in comparison to cells in the OFF state. Values represent ratios of σ: mean FI. The error bar denotes the 95% confidence interval around the mean of all measurements. Number of replicates N for each bar shown in (B) is 8, with in total 310 considered cells. Detailed information on analyzed cell numbers can be found in the S1 Data file. (TIF) [file pone.0227249.s006.tif]

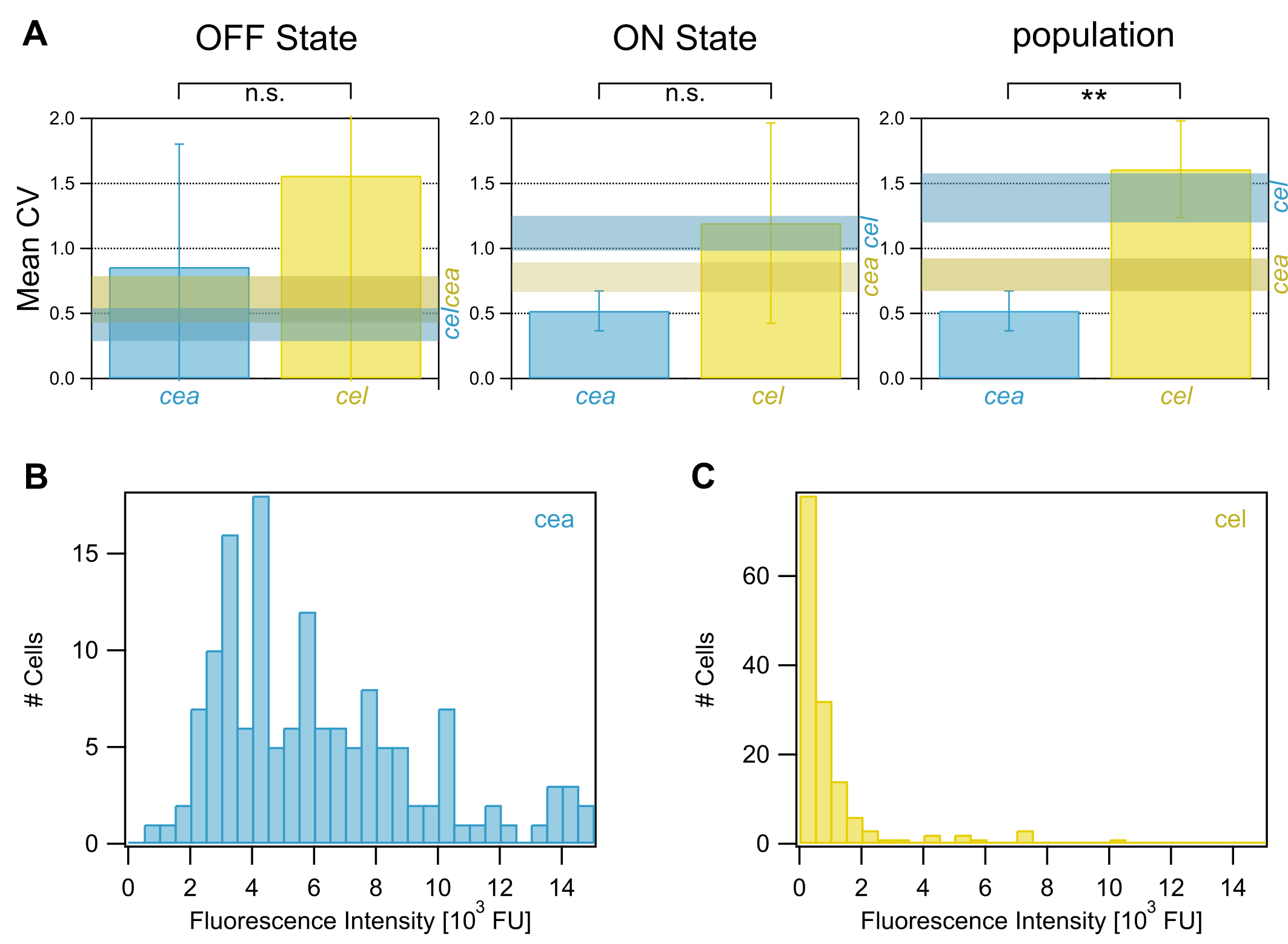

Supplement: S4 Fig — A) Noise in cea (cfp) expression is given in blue, noise in cel (yfp) expression is given in yellow. Transparent areas yellow (YFP) and in blue (CFP) indicate the noise values for the S strain (cea (yfp), cel (cfp), Materials and methods, S1 Table) Basal noise of cells in the OFF state (left). Noise of cells in the ON state (middle). Population noise (right). Significance levels of the corresponding SFLIP distributions: n.s. no significant difference; **: p<0.01. B/C) Histograms of fluorescence intensity distribution of the entire population FI at 270min for B) cea (cfp) and C) cel (yfp) of the SFLIP mutant for all measurements. The error bar denotes the 95% confidence interval around the mean of all measurements. Number of replicates N for each bar shown in (A) is 3, with 143 considered cells in total. Detailed information on analyzed cell numbers can be found in the S1 Data file. (TIF) [file pone.0227249.s007.tif]

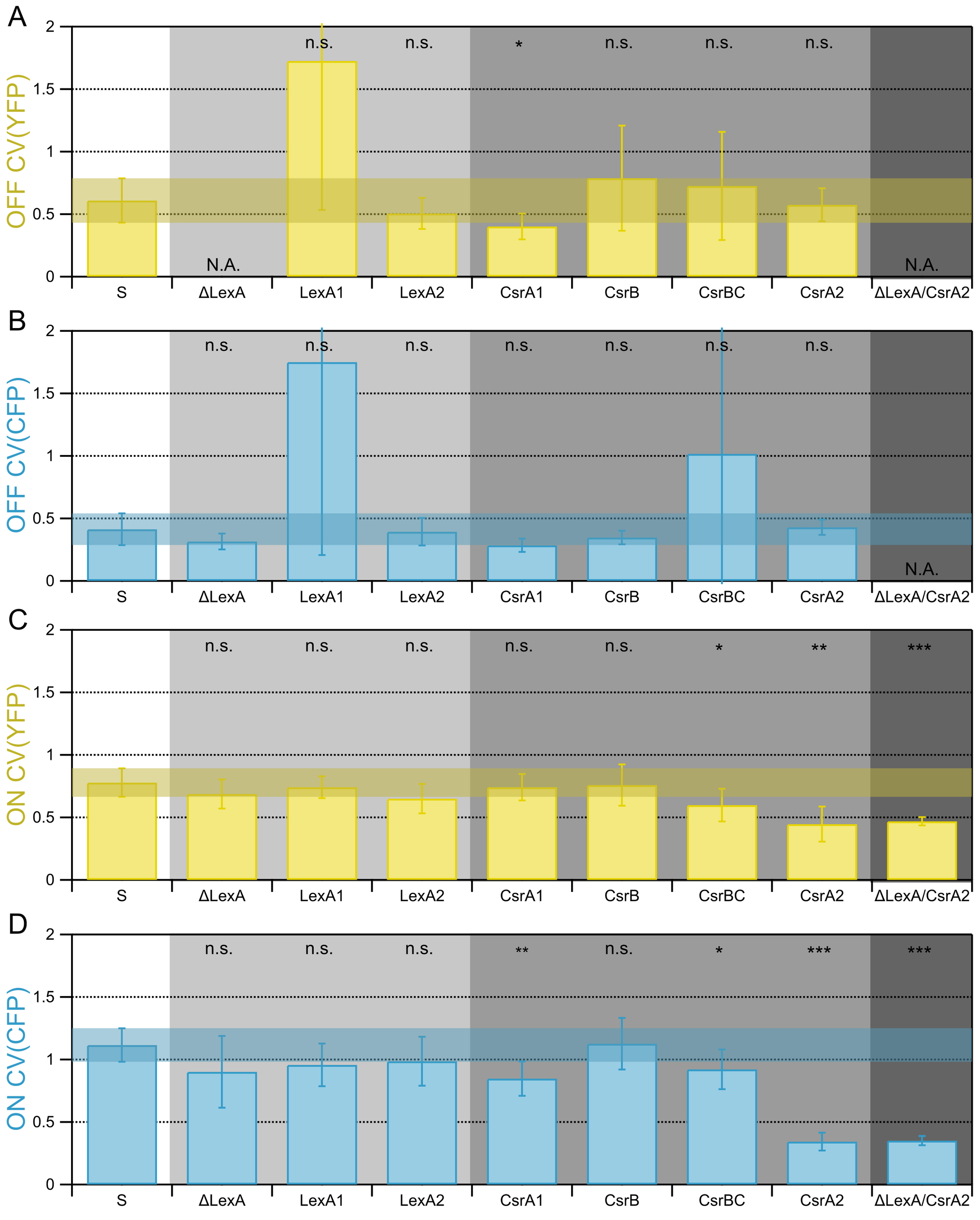

Supplement: S5 Fig — Coefficient of variation (CV) of YFP gene expression is shown in yellow, of CFP expression in blue. A,B) Noise of genes in bacterial cells in the OFF state. C,D) noise of genes in bacterial cells in the ON state. Background color indicates genetic changes in transcriptional (T, light grey) or post-transcriptional regulation (PT, medium grey), or both (T and PT, dark grey). Transparent colored regions (YFP, yellow and CFP, blue) indicate the CV values (with the corresponding error) of the S strain for better comparability between data sets. Significance levels are set as n.s. no significant difference; *: p<0.05; **: p<0.01; ***: p<0.001 and represent the comparison to the S strain. The error bar denotes the 95% confidence interval around the mean of all measurements. Number of replicates N for each strain is S: 8, ΔLexA: 9, LexA1: 11, LexA2: 10, CsrA1: 9, CsrB: 6, CsrBC: 7, CsrA2: 7, ΔLexA/CsrA2: 7. Total number of considered cells X: S: 310, ΔLexA: 301, LexA1: 431, LexA2: 382, CsrA1: 434, CsrB: 314, CsrBC: 312, CsrA2: 348, ΔLexA/CsrA2: 247. Detailed information on analyzed cell numbers (ON/OFF state, YFP/CFP) can be found in the S1 Data file. (TIF) [file pone.0227249.s008.tif]

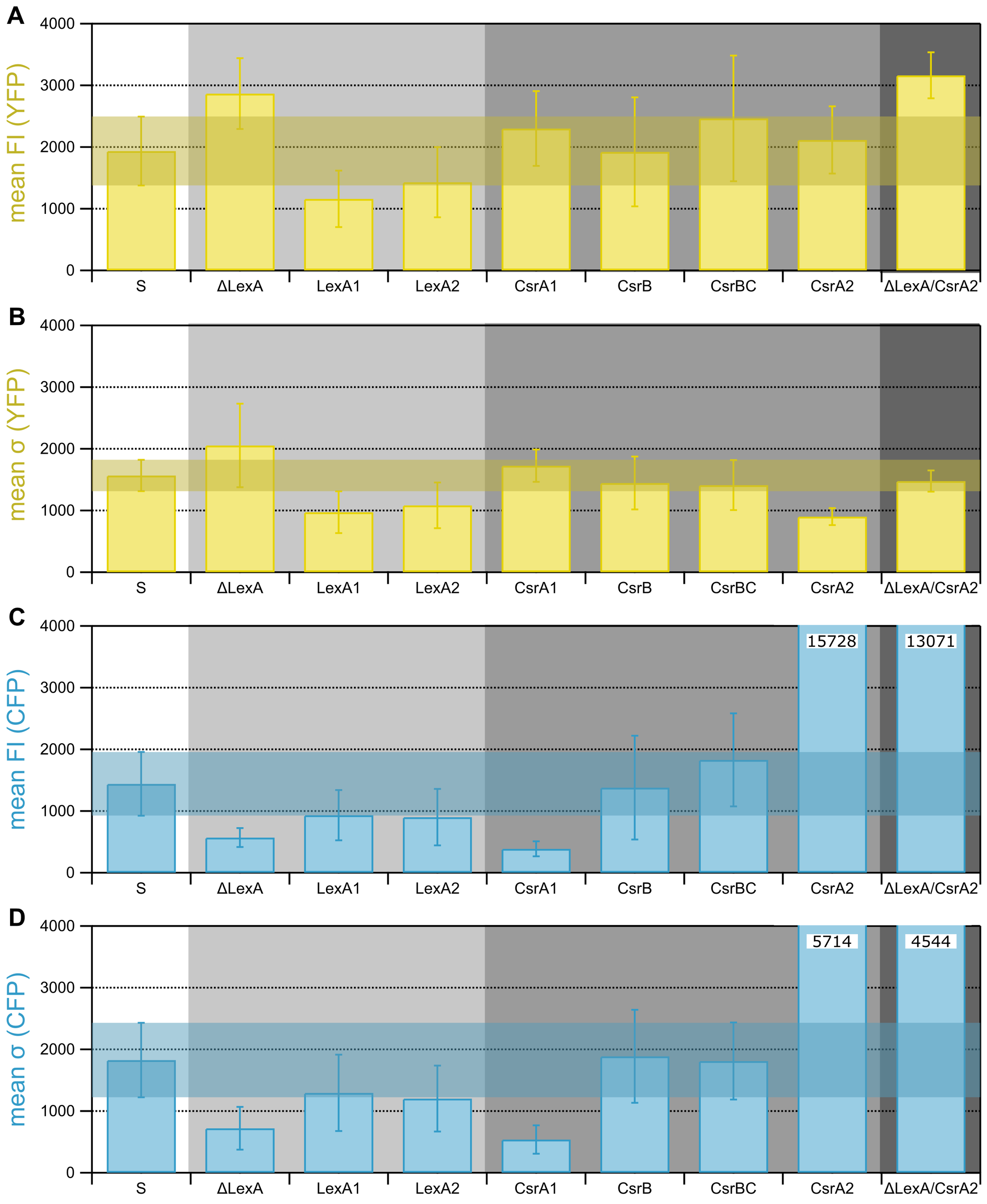

Supplement: S6 Fig — A) Mean FI and B) mean σ of YFP gene expression are shown in yellow. C) Mean FI and D) mean σ of CFP expression are shown in blue. Background color indicates genetic changes in transcriptional (T, light grey) or post-transcriptional regulation (PT, medium grey), or both (T and PT, dark grey). Transparent colored regions (YFP, yellow and CFP, blue) indicate the corresponding values (with error) of the S strain for better comparability between data sets. Values highlighted in white are the FI and σ values for the corresponding bars as they were much higher than those of the other mutants and axis was cut for better visibility. The error bar denotes the 95% confidence interval around the mean of all measurements. Number of replicates N for each strain is S: 8, ΔLexA: 9, LexA1: 11, LexA2: 10, CsrA1: 9, CsrB: 6, CsrBC: 7, CsrA2: 7, ΔLexA/CsrA2: 7. Number of considered cells X: S: 310, ΔLexA: 301, LexA1: 431, LexA2: 382, CsrA1: 434, CsrB: 314, CsrBC: 312, CsrA2: 348, ΔLexA/CsrA2: 247. Detailed information on analyzed cell numbers can be found in S3 Table. (TIF) [file pone.0227249.s009.tif]

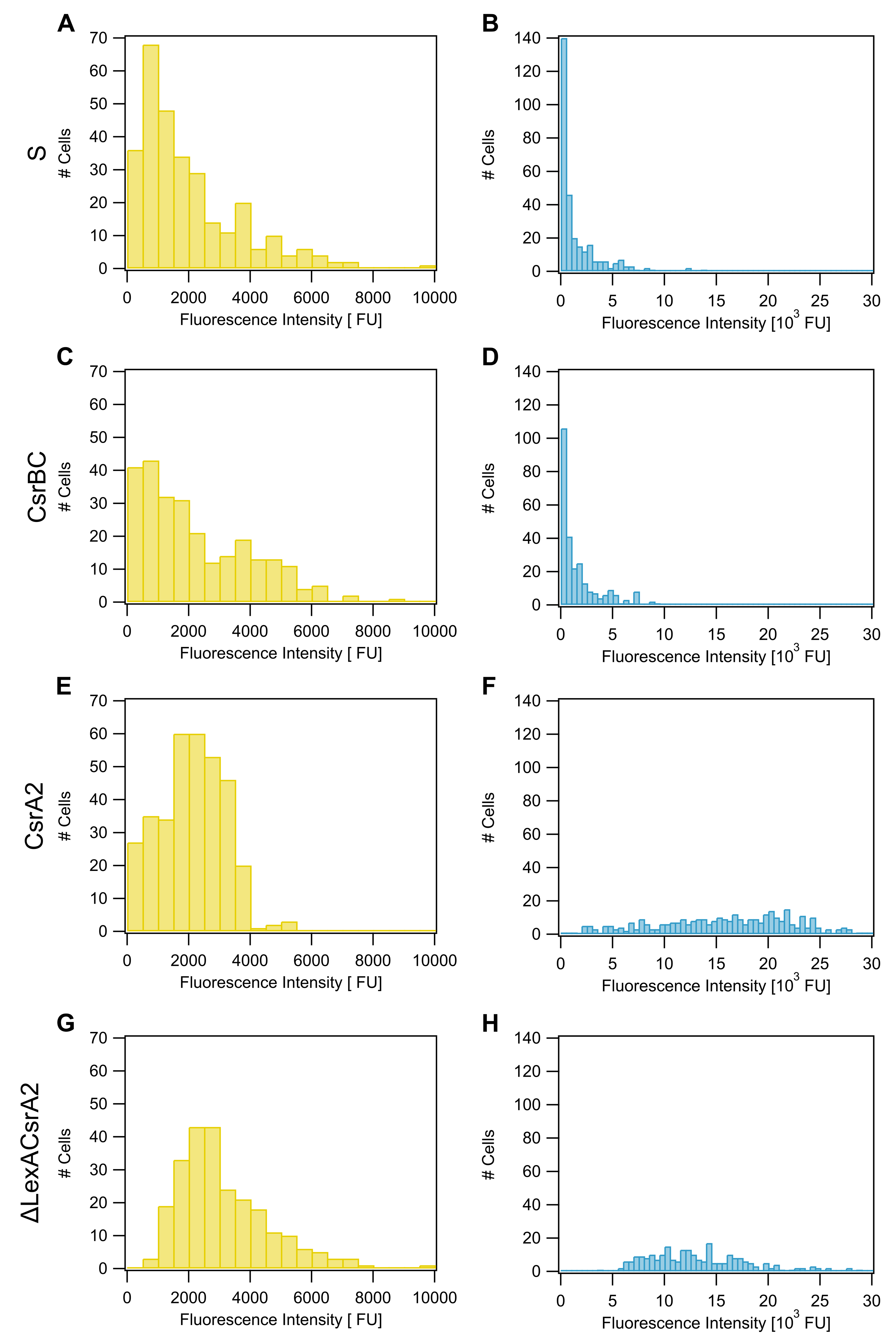

Supplement: S7 Fig — A,C,E,G) YFP expression and B,D,F,H) CFP expression histograms for all measured cells at three MitC concentrations are depicted in yellow and blue respectively. Histograms are corresponding to the population noise state of all cells at 270min of the measurement. (TIF) [file pone.0227249.s010.tif]

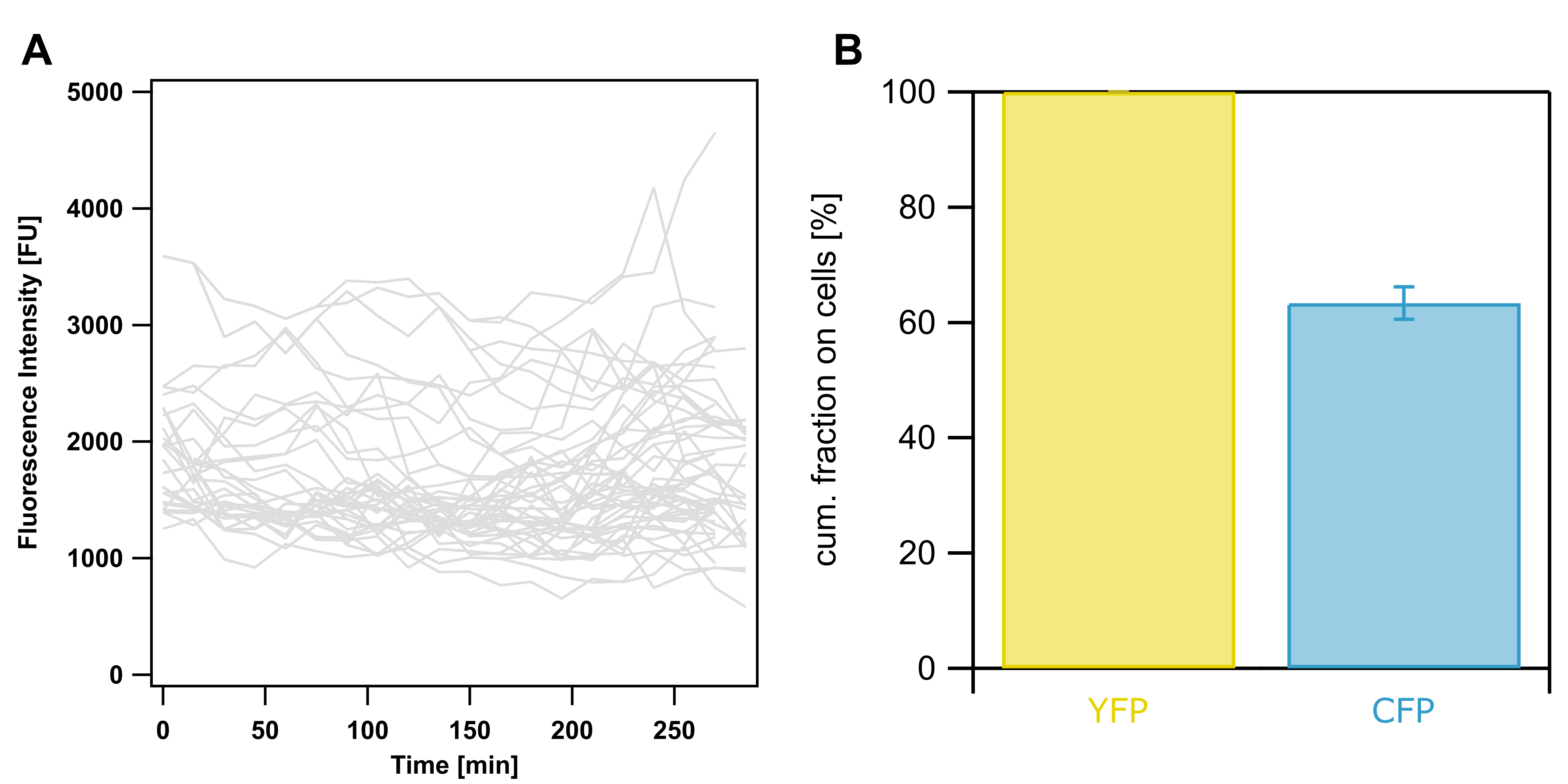

Supplement: S8 Fig — A) Single traces of 50 cells expressing YFP over time (grey). B) Fraction of cells expressing YFP and CFP in Δ LexA. Please note that the long mRNA including the cfp gene (Fig 1) is less frequent. Furthermore, the cfp gene underlies additional post-transcriptional regulation by CsrA. Hence, the fraction of cells expressing cfp is not 100%. The error of the cumulative fraction of cells in the ON state is given by the standard error of the mean (SEM) with the error bar. (TIF) [file pone.0227249.s011.tif]

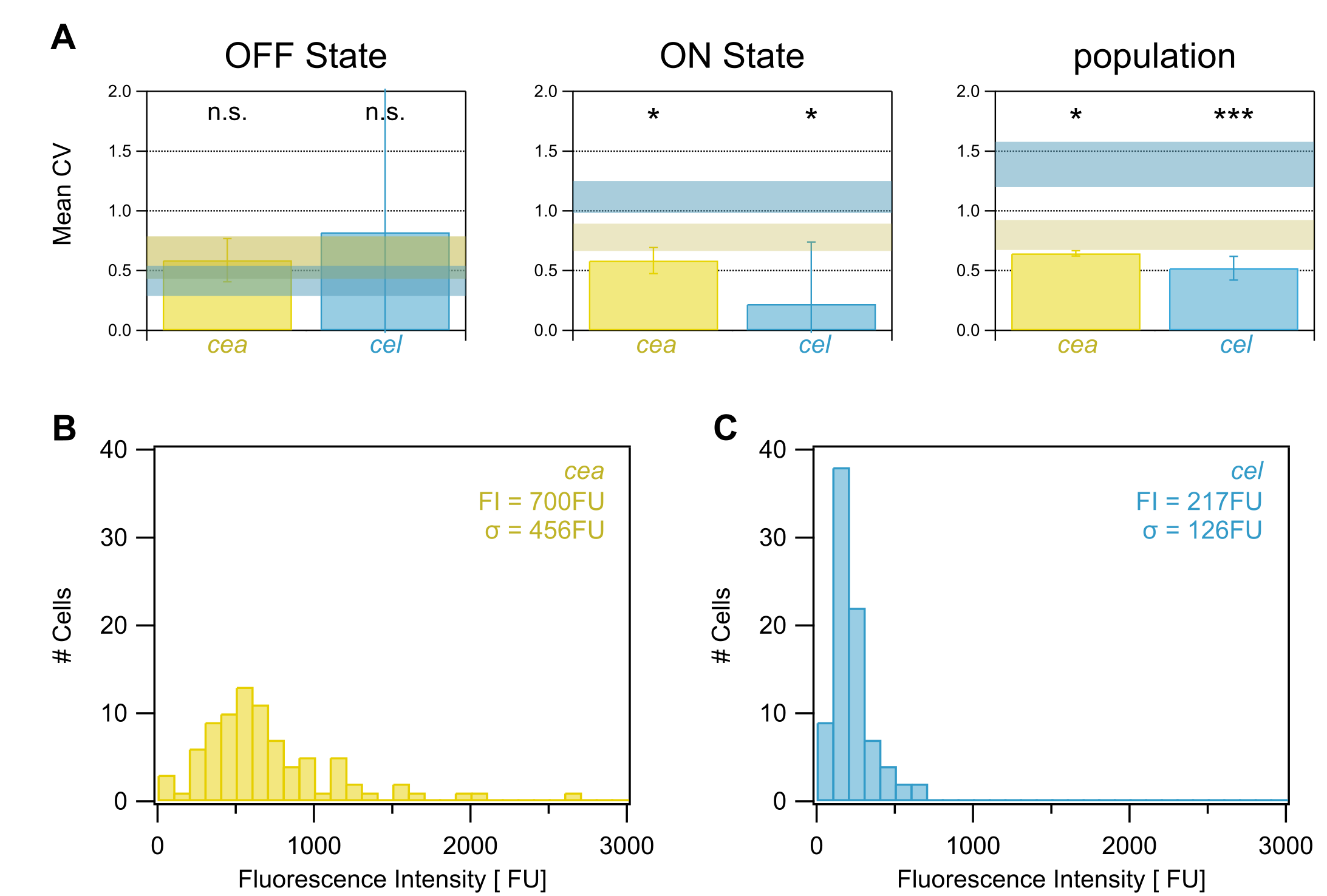

Supplement: S9 Fig — A) Noise in YFP expression is given in yellow, noise in CFP expression is given in blue. Transparent areas yellow (YFP) and in blue (CFP) indicate the noise values for the S strain with high plasmid copy number (~ 55 copies, Materials and methods, S1 Table) Basal noise of cells in the OFF state (left). Noise of cells in the ON state (middle). Population noise (right). Significance levels of the corresponding SREP2 distribution compared to the S strain: n.s. no significant difference; *: p<0.05; **: p<0.01; ***: p<0.001. B/C) Histograms of fluorescence intensity distribution of population FI at 270min on B) cea (yfp) and C) cel (cfp) of the SREP2 strain for all measurements. The error bar denotes the 95% confidence interval around the mean of all measurements. Number of replicates N for each bar shown in (A) is 3, with 93 considered cells in total. Detailed information on analyzed cell numbers can be found in the S1 Data file. (TIF) [file pone.0227249.s012.tif]
